# Development of Orthogonal Linear Separation Analysis (OLSA) to Decompose Drug Effects into Basic Components

**DOI:** 10.1038/s41598-019-38528-4

**Published:** 2019-02-12

**Authors:** Tadahaya Mizuno, Setsuo Kinoshita, Takuya Ito, Shotaro Maedera, Hiroyuki Kusuhara

**Affiliations:** 1 Graduate School of Pharmaceutical Sciences, the University of Tokyo, Bunkyo-ku, Tokyo 113-0033 Japan; 2 ProMedico Co., Ltd., Ota-ku, Tokyo 143-0023 Japan

## Abstract

Drugs have multiple, not single, effects. Decomposition of drug effects into basic components helps us to understand the pharmacological properties of a drug and contributes to drug discovery. We have extended factor analysis and developed a novel profile data analysis method: orthogonal linear separation analysis (OLSA). OLSA contracted 11,911 genes to 118 factors from transcriptome data of MCF7 cells treated with 318 compounds in a Connectivity Map. Ontology of the main genes constituting the factors detected significant enrichment of the ontology in 65 of 118 factors and similar results were obtained in two other data sets. In further analysis of the Connectivity Map data set, one factor discriminated two Hsp90 inhibitors, geldanamycin and radicicol, while clustering analysis could not. Doxorubicin and other topoisomerase inhibitors were estimated to inhibit Na^+^/K^+^ ATPase, one of the suggested mechanisms of doxorubicin-induced cardiotoxicity. Based on the factor including PI3K/AKT/mTORC1 inhibition activity, 5 compounds were predicted to be novel inducers of autophagy, and other analyses including western blotting revealed that 4 of the 5 actually induced autophagy. These findings indicate the potential of OLSA to decompose the effects of a drug and identify its basic components.

## Introduction

The response to a drug can be a complex of the entire biological responses to the perturbagen and multiple responses in living systems. Not all the effects of a drug are fully discovered by researchers or developers. Therefore, to separate the complex effects of a drug into basic components is a prerequisite for a deep understanding of the pharmacological properties of drugs, which contributes to drug screening, drug repositioning, prediction of toxicity, and other properties.

Omics has made a great impact on biology since its emergence^1^. The comprehensive nature of the methodology can translate the biological information of a sample into numeric data, and because of this characteristic, omics data are also called a profile. This quality of omics affords us mathematical approaches to comprehend the sample characteristics and are referred to as profile data analysis, or simply profiling. A substantial number of profiles have been accumulated and many analysis methods have been devised^2,3^.

Notably, the Connectivity Map (CMap) project initiated by the Broad Institute greatly contributed to the field^4,5^. In the project, dozens of microarray data analysing cells treated with low molecular weight compounds were collected in the same platform. The concept is simple: a “signature” is simply defined by up- and down-regulated genes responding to a perturbagen and the signatures can be compared to identify drugs with similar effects^4^. One of the essential features of this approach is not focusing on each gene, but on the relationship of genes described as a gene pattern, or signature. There exist phenotypes that cannot be identified by the analysis of each gene^6^. Another curious characteristic of CMap is that it does not depend on existing knowledge, which distinguishes this approach from gene ontology (GO) analysis or pathway analysis^7,8^. Use of existing knowledge in profiling is effective in reducing noise in profile data, while it restricts the capacity of analysis within the known. Analyses with CMap even use information unrecognized by researchers and therefore have the potential to reveal new discoveries. Many studies using CMap have succeeded in drug repositioning^9–11^.

Considering the complex effect of a drug, we began to investigate whether it is possible to decompose it into basic components described by variable patterns using profile data analysis, particularly in an unsupervised way, and focused on factor analysis (FA). FA decomposes a data matrix based on standard deviation, is well established in various fields, and is also used in omics data analysis^12,13^. Many studies accomplish dimension reduction and feature extraction of omics data to classify or investigate the similarity of samples with FA^12,13^. However, to our knowledge, there are no studies that employ FA to separate the effects of a drug and extract the more basic components.

Among the several types of FA, the combination of principal component analysis (PCA) and following varimax rotation has been used extensively in the history of FA. The characteristics are that the new indicators (factors in FA) comprising the original variables are mutually orthogonal^14^. We consider that the effect of a perturbagen can be described to some degree by a linear combination of more basic effects, while the remaining parts are non-linearly integrated and not separable^15^. Notably, linear separation enables us to approach the molecular mechanism behind the composition using an omics data matrix in which the new indicators generated are easier to comprehend than those obtained by non-linear separation or machine learning^16^.

A concern of using FA with the principal component method in profiling is that the centroid in the novel co-ordinate space has no biological meaning and varies among data sets, which means that the obtained factors (vectors) in such a situation may not correspond to consistent biological meanings. To address that concern, we have extended FA in the following two points: to use response profiles and to add the mirror data set to the original in FA. We call the profile data analysis with the simply modified FA orthogonal linear separation analysis (OLSA). Here, we report the performance and possibility for OLSA to separate a perturbagen effect into basic components by analysing transcriptome profiles.

## Results

### The concept of OLSA of profile data

**Figure 1:**
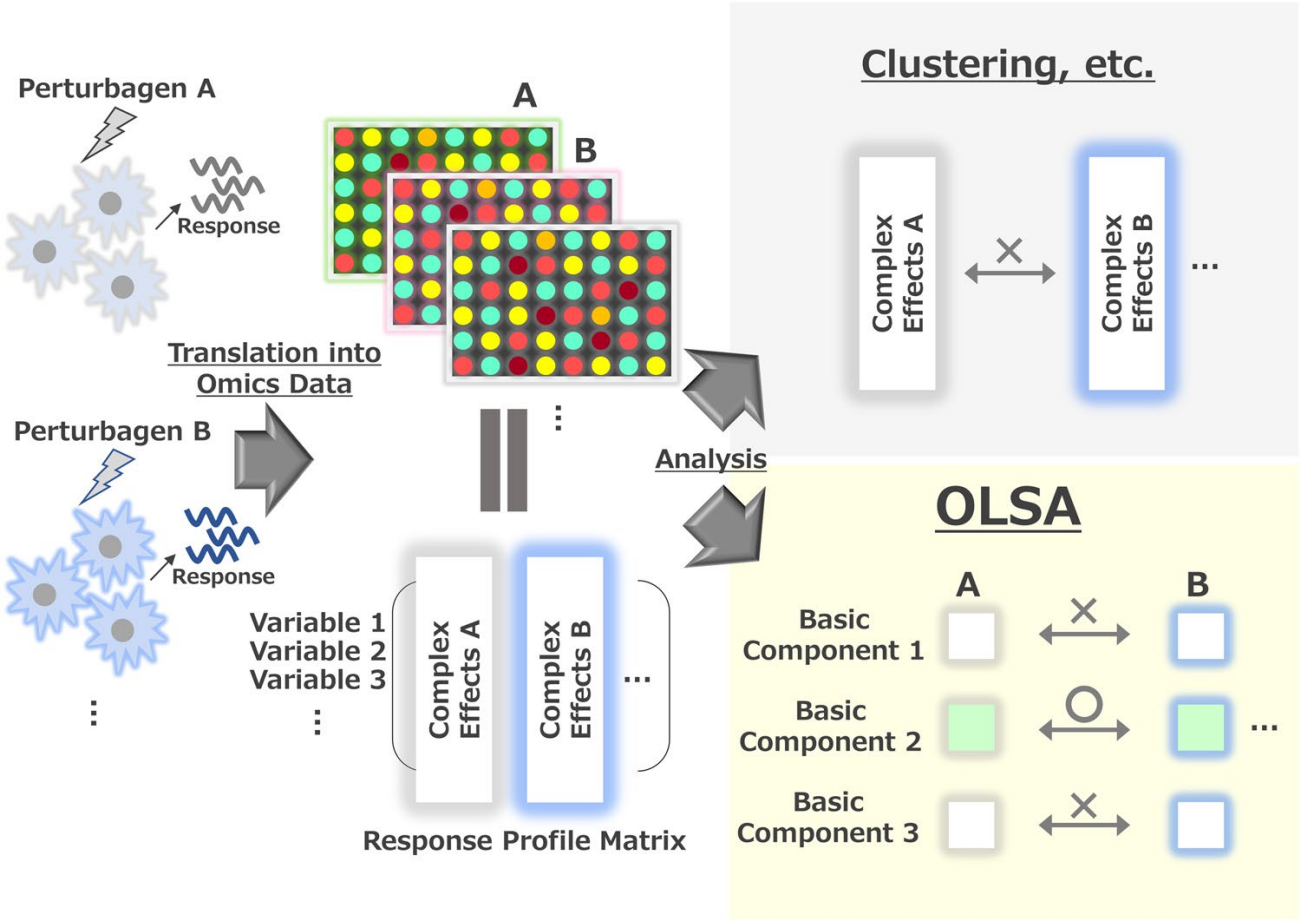
The concept of orthogonal linear separation analysis of profile data. Illustration of OLSA application to response-profile data.

The workflow and concept of OLSA of a response-profile matrix are shown in Fig. 1a and Supplementary Fig. S1. Here, we define a “response-profile matrix” as a matrix with variables (e.g., gene expression change) in rows and samples in columns. An element in a response-profile matrix is a value representing a change of expression of a factor, such as a log fold change or *z* score versus control. By converting the raw expression values of profile data into response values, the origin of the response data space represents the control treatment or no stimulation. One of the characteristics of OLSA is the use of a mirror data set (point-symmetric to the analysed response-profile data). Considering the reversibility of biological responses, the mirror data set represents the assumed antagonizing or reverse responses to the original data as a virtual data set. FA of the combined data set enables us to approximate mathematically the novel co-ordinate space centroid to the origin of the original data space, where the variables are biologically relevant. Therefore, we can expect that the generated factors have consistent biological meanings. By employing OLSA, a response-profile matrix is described by the product of a response-vector matrix, a response-score matrix, and a total strength matrix, corresponding to the eigenvector matrix, the loading matrix, and a diagonal matrix of the L2-norm used for intensity correction (Supplementary Fig. S1). In OLSA, each response vector constituting a response-profile matrix is composed of a gene list with values and corresponds to “factor” in conventional FA with the principal component method, which describes an array of response-profile data with a linear combination of factors summarizing the original high-dimensional data and helps us to comprehend the biological information of a profile data by investigating each separated factor (Supplementary Fig. S1).

We assumed that the factors isolated by OLSA are biologically relevant and the indicators of “basic” biological responses that constitute the original complex effect of a perturbagen. In the following, we confirmed that the factors generated with OLSA are biologically relevant and then investigated whether the application of this method contributes to understanding the effects of the perturbing drugs.

### Confirmation of biological meanings of the generated factors with OLSA

#### Cellular responses in MCF7 cells treated with 370 perturbagens

**Figure 2:**
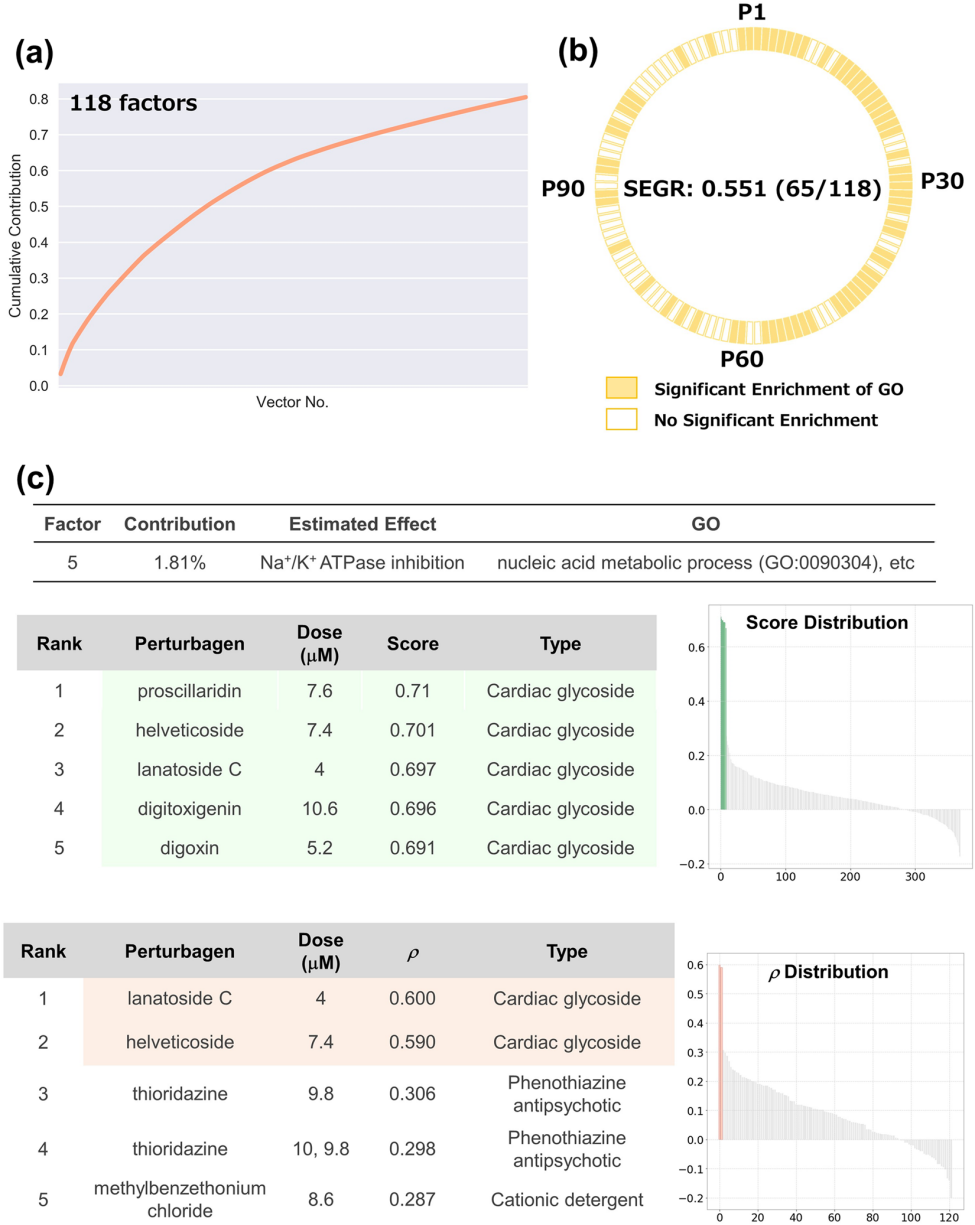
Analysis of cellular responses in MCF7 cells treated with 370 perturbagens. (**a**) The cumulative contribution curve of the factors contracting the training data set. The contribution of each factor to the total deviation was calculated and arranged in descending order. The cumulative contribution was calculated from the top and plotted. (**b**) Plot of the factors whose main constituents exhibit significant enrichment of gene ontology. Genes constituting a response vector were sorted by the square of each value. The top 1% of genes were subjected to GO (biological process) analysis using the Enrichment analysis of the Gene Ontology Consortium. Factors annotated with significant enrichment of GO after multiple-testing corrections (Benjamini–Hochberg method, α < 0.05) are depicted in yellow-filled squares. SEGR, significant enrichment of GO. (**c**) Analysis of P5 factor. P5 factor (the factor with the 5^th^ highest contribution) scores and rho (*ρ*) of all compounds are arranged in descending order and plotted on the “Score Distribution” graph and “*ρ* Distribution” in each data set, respectively (upper, training; lower, test). Green or light salmon in the graph indicates a “cardiac glycoside”. The rank, name, dose, and score of the top 5 compounds are shown.

We started with an analysis of the response-profile matrices obtained from CMap to verify OLSA. We analysed the profile data set investigating the cellular responses of MCF7 cells treated with 318 compounds as a training set (Supplementary Fig. S2). We subjected the data to varimax rotation and analysed 118 vectors, accounting for 80% of cumulative contribution (Fig. 2a). To obtain insight into the biological relevance of the factors, we used GO analysis of the genes that had a large absolute value in a factor and that mainly characterised the factor. Genes constituting a factor were sorted by their contribution to the factor and the top 1% of genes were subjected to GO analysis. Statistical significance was judged with enrichment analysis provided by a GO consortium, which conducts Fisher’s exact test between a focusing gene list and a gene list constituting a GO. There were 65 factors with significant enrichment of GO, and the ratio of such factors (hereafter termed significant enrichment of GO ratio, SEGR) to the total was 0.551 (65/118) (Fig. 2b).

Given that the generated factors are biologically relevant, the gene patterns are supposed to be conserved in another data set and are useful in identifying compounds with a focused cellular response. To verify this supposition, we characterized several factors using a detailed literature survey and subsequently calculated the Spearman’s correlations between the selected factors and another data set. For the test data set, we employed a profile set comprising 122 transcriptome data analysing PC3 cells treated with 104 compounds provided by CMap (Supplementary Fig. S2).

The high-scoring compounds in the factor with the 5^th^ highest contribution (hereafter P5 factor) were cardioglycosides and all 8 cardioglycosides in the data set were ranked in the top 9 (Fig. 2c, upper panel). Both cardioglycosides in the test set, lanatoside C and helveticoside, were ranked in the top 2 of the compound list sorted by Spearman’s correlation coefficients, supporting that the P5 factor as including cardioglycoside effects such as Na^+^/K^+^ ATPase inhibition (Fig. 2c, lower panel).

Similarly, several factors were clearly connected to biologically relevant responses and the following are particularly interesting (Supplementary Fig. S2). Flavonoids with a similar structure dominated the top 4 of the P35 factor, while the gene pattern exhibited no enriched GO. The P76 factor was associated with ion modulation responses although the factor contribution was quite low (0.3% of the total). The positive high-scoring compounds in the P7 factor were oestrogens, while the negative-scoring compounds were anti-oestrogens, which was consistent with the CMap results and suggests that the signs of the response scores correspond to the direction of basic cellular responses^4^.

Together, these data support that factors separated linearly by OLSA reflect cellular responses in the CMap data set.

#### Cellular responses in HepG2 cells treated with genotoxic compounds

**Figure 3:**
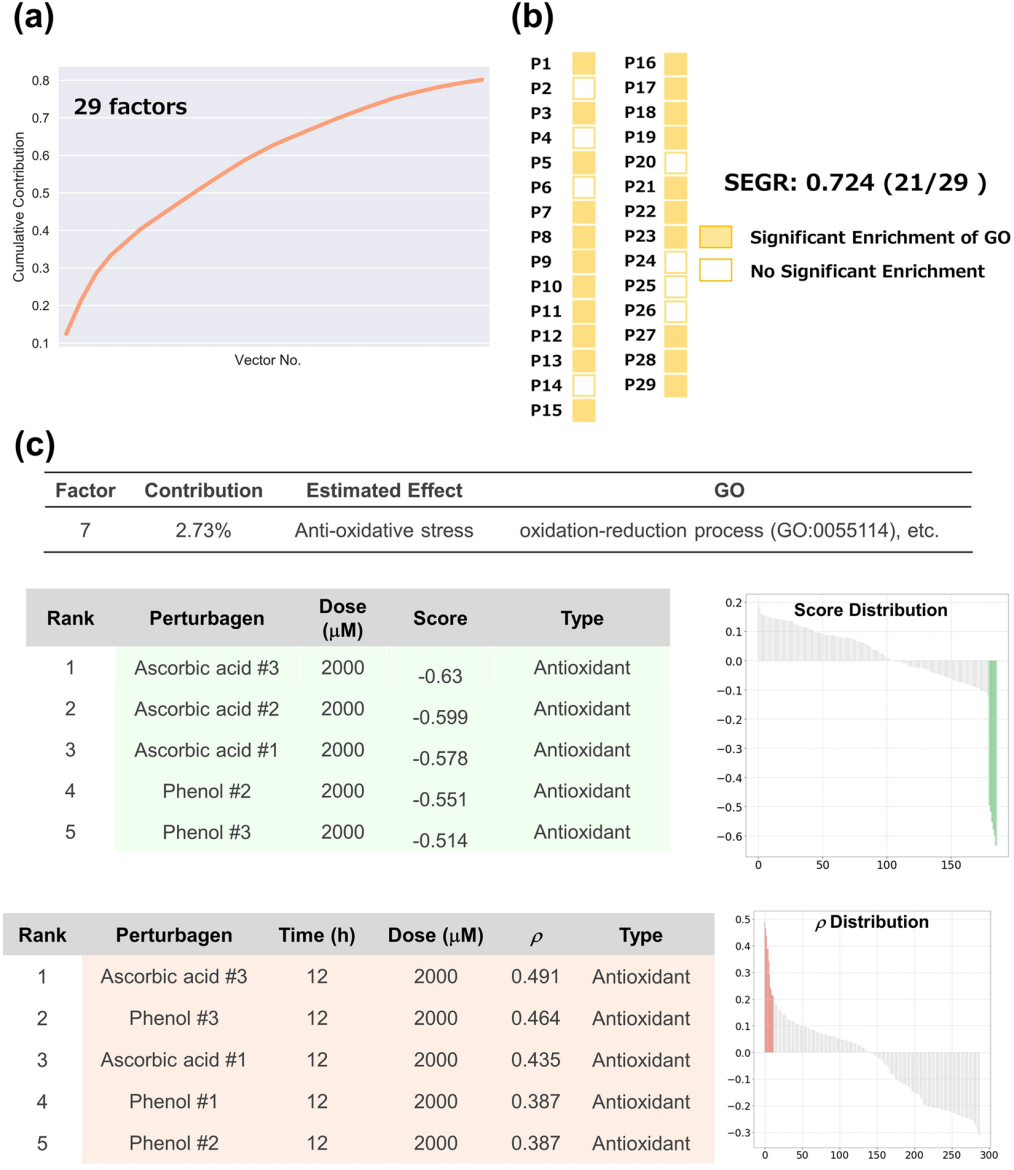
Analysis of cellular responses in HepG2 cells treated with 62 genotoxic compounds. (**a**) The cumulative contribution curve of the factors comprising the training data set. The contribution of each factor to the total deviation was calculated and arranged in descending order. The cumulative contribution was calculated from the top and plotted. (**b**) Plot of the factors whose main constituents exhibit significant enrichment of gene ontology. Genes constituting a response vector were sorted by the square of each value. The top 1% of genes were subjected to GO (biological process) analysis using the Enrichment analysis of Gene Ontology Consortium. Factors annotated with significant enrichment of GO after multiple-testing corrections (Benjamini–Hochberg method, α < 0.05) are depicted in yellow-filled squares. SEGR, significant enrichment of GO. (**c**) Analysis of P7 factor. P7 factor scores and rho (*ρ*) of all compounds are arranged in descending order and plotted on the “Score Distribution” graph and “*ρ* Distribution” in each data set, respectively (upper, training; lower, test). Green in the graph indicates ascorbic acid and light salmon indicates phenol. The rank, name, dose, and score of the top 10 compounds are shown. “–” and “#” indicate not investigated in the literature survey and the number of biological replicates, respectively.

To investigate whether OLSA is effective for data sets other than CMap, we applied the method to data obtained from a public transcriptome database. Magkoufopoulou *et al*. investigated the transcriptome profiles of HepG2 cells treated with 158 genotoxic compounds and obtained 474 transcriptome data^17^. We employed these data and separated them into two groups: the data of 24 h treatment for training and the data of 12 and 48 h for the test (Supplementary Fig. S3). The data in each set were converted into response-profile matrices, and the processed training data set was subjected to OLSA. The analysis generated 29 factors from 186 transcriptome data up to 80% cumulative accumulation (Fig. 3a). GO analysis revealed that 21 of 29 (SEGR; 0.724) factors exhibited significant enrichment of GO (Fig. 3b).

In a detailed investigation of individual factors, several factors were clearly connected to biologically relevant responses. For instance, the negative-scoring compounds in the P7 factor were dominated by ascorbic acid and phenol, and both of them had antioxidant properties in common (Fig. 3c, upper panel)^18^. The test set was validated by calculating Spearman’s correlation coefficients between the gene pattern and the test set. Both compounds exhibited high values regardless of treatment time. Moreover, one of the GOs significantly enriched in the factor is “oxidation-reduction process (GO:0055114)”, supporting the consistency of the biological meaning of the factors.

Similarly, the P15, P28, and P29 factors were suggested to include P450 modulation, aryl hydrocarbon receptor stimulation, and interferon I stimulation effects, respectively (Supplementary Fig. S3)^19,20^. These results indicate that OLSA application is not restricted to well-aligned data sets such as those provided by CMap.

#### Inflammatory responses in macrophages

We investigated the capacity of OLSA in a response-profile matrix composed of relatively few data. Raza *et al*. investigated transcriptional networks in murine macrophages treated with several inflammatory stimulants at various time points by analysing the transcriptome data set composed of 60 data with 30 perturbagens (2 biological replicates each)^21^. We separated the data set into training and test sets: the data of bone marrow derived macrophages (BMDM) from BALB/c mice for the training and BMDM from C57/BL6 mice for the test (Supplementary Fig. S4). The training data set was processed to obtain a response-profile matrix and subjected to OLSA. We obtained 15 factors and the SEGR was 0.33 (5/15) (Supplementary Fig. S4).

**Figure 4:**
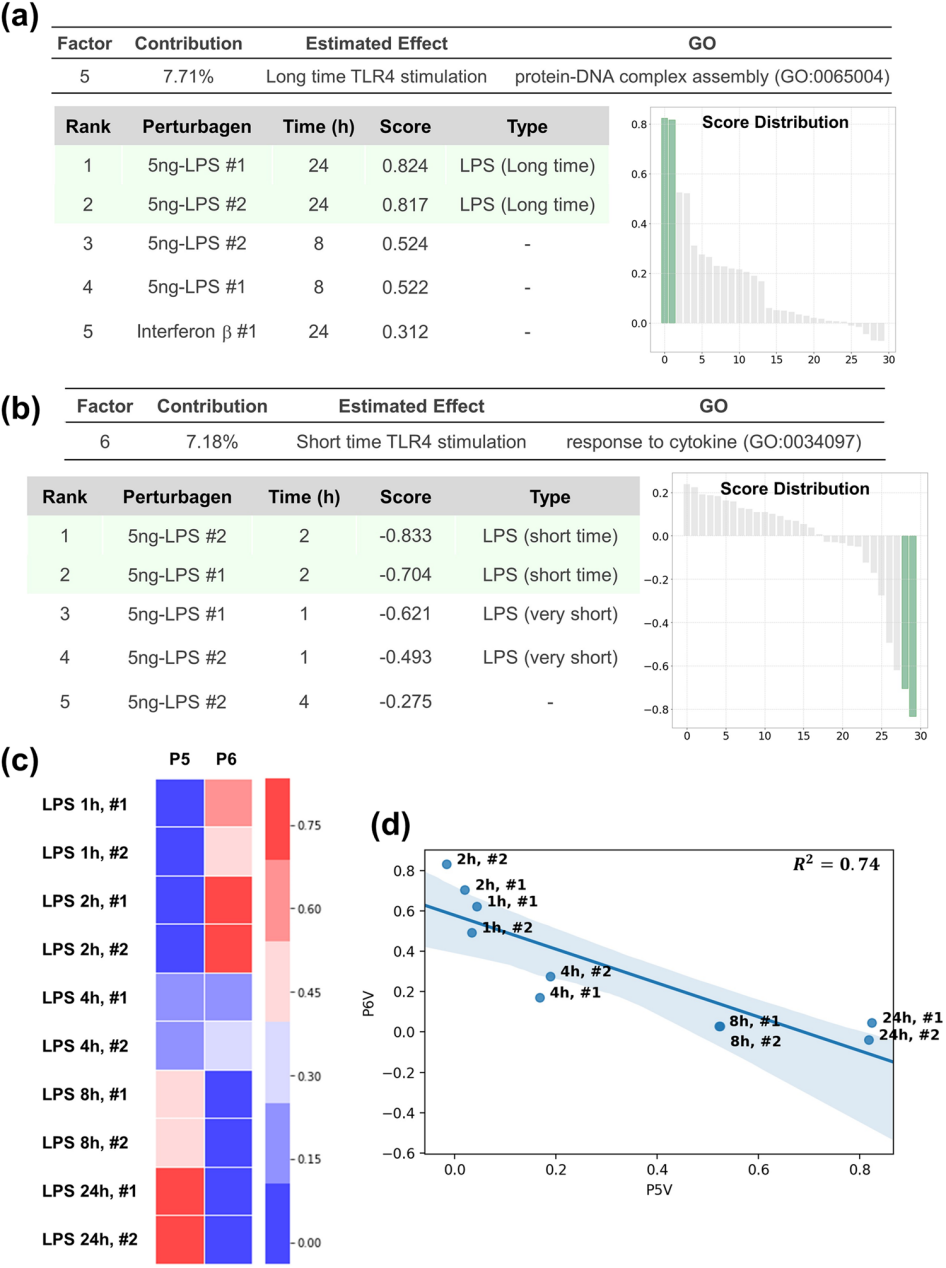
Analysis of inflammatory responses in macrophages. (**a**) Analysis of the P5 factor. P5 factor scores and rho (*ρ*) of all compounds are arranged in descending order and plotted on the “Score Distribution” graph and “*ρ* Distribution” in each data set, respectively (upper, training; lower, test). Green or light salmon in the graph indicates 24-h LPS treatment. The rank, name, dose, and score of the top 10 treatments are shown. “–”, “#”, and “5 ng-” indicate without 24-h LPS treatment, the sample number of biological replicates, and 5 ng/mL treatment, respectively. (**b**) Analysis of the P6 factor using the method described in a. Green or light salmon in the graph indicates 2-h LPS treatment. The rank, name, dose, and score of the top 10 treatments are shown. “–” indicates no 2-h LPS treatment. (**c**) Heatmap comparing the scores of P5 and P6 factors. 1 h, …, 24 h and #1, #2 indicate 1 h-, …, 24 h treatment and the number of biological replicates, respectively. (**d**) Scatter plot of the scores of P5 and P6 factors. The blue line and area indicate the regression line and the 95% confidence interval. *R*^2^, the coefficient of determination.

Lipopolysaccharide (LPS), a well-known endotoxin, exhibits various properties as an inflammatory stimulant by binding to toll-like receptor 4 and the effect varies from one hour to another in macrophages^22^. Both replicates of LPS-24-h and 2-h treatment were ranked in the top 2 of the perturbagens list sorted by the P5 and P6 factors, respectively, and the conservation of the gene patterns in another data set was confirmed (Fig. 4a,b and Supplementary Fig. S4). Notably, the scores of the two factors exhibited clear inverse correlation with regard to time points (Fig. 4c,d), which supports that OLSA succeeded in extracting time-dependent responses of LPS as reported^22,23^. It should be noted that LPS treatment for 1 h did not correlate with the P6 factor in the test data set although the treatment was “short”. One explanation is that an hour is too short to activate the transcriptional network constituting the P6 factor.

The responses to interferon β and γ treatment for 24 h seemed to be included in the P10 and P15 factors, respectively, although we were not able to validate the responses in another data set because of a lack of data (Supplementary Fig. S4). These results indicate that OLSA works in the analysis of a response-profile matrix composed of relatively small transcriptome data.

### Application of OLSA in understanding the effects of drugs

#### Decomposition of Hsp90-inhibitor effect

Next, we investigated whether OLSA contributes to an understanding of the effects of drugs by analysing the CMap data set.

**Figure 5:**
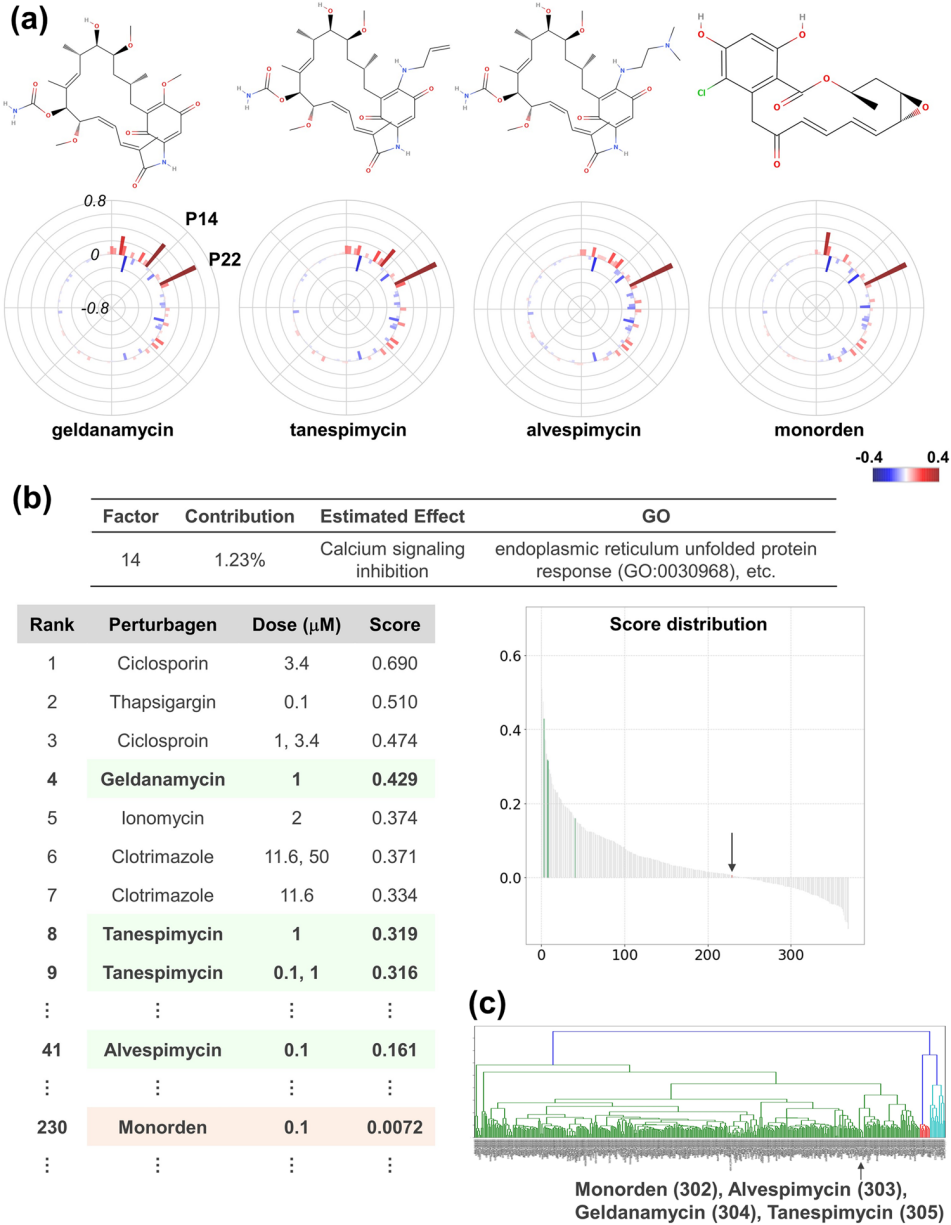
Decomposition of Hsp90-inhibitor effect. (**a**) Structures and response scores of Hsp90 inhibitors. Structures were obtained from MolView (http://molview.org/). Response scores are plotted as a bar chart in polar co-ordinates with heatmap. (**b**) Analysis of the P14 factor. P14 factor scores of all compounds are arranged in descending order and plotted on the “Score Distribution” graph. Green with an arrow in the graph indicates geldanamycin-type inhibitors and light salmon with an arrow indicates monorden. The rank, name, dose, and score are shown. (**c**) Clustering analysis of MCF7 cells data set of CMap. The MCF7 cells data set of CMap was subjected to clustering analysis with the Ward method. An arrow indicates the cluster where Hsp90 inhibitors belong. The numbers following the compound names indicate the ordinal numbers from the left.

Hsp90 inhibitors are potent anti-cancer reagents in development^24^. The first compound identified in this class of inhibitor is geldanamycin, as found in *Streptomyces hygroscopicus*^24^, followed by the synthesis of tanespimycin and alvespimycin via lead optimization^25^. Monorden was found from *Pochonia chlamydosporia* and is structurally distinct from geldanamycin (Fig. 5a)^25^.

In the OLSA result, these Hsp90 inhibitors (geldanamycin, tanespimycin, alvespimycin, and monorden) exhibited high scores in the P22 factor, which suggests that this factor includes an Hsp90 inhibition effect (Supplementary Fig. S5). Interestingly, there also exists a difference among them: geldanamycin and tanespimycin exhibited high scores in the P14 factor, while those of alvespimycin and monorden were not high and almost zero, respectively (Fig. 5b and Supplementary Fig. S5). The compounds that ranked high in the P14 factor score list were cyclosporin (a calcineurin inhibitor), thapsigargin (an ER calcium depleter), and ionomycin (a calcium ionophore), and they indicate that the factor includes calcium signalling inhibition. Therefore, based on the P14 score, geldanamycin and tanespimycin are considered to have a high inhibitory effect of calcium signalling while the effect of alvespimycin and monorden is predicted to be mild and low, respectively. Indeed, Chang *et al*. elucidated the difference between geldanamycin and monorden and reported that only the former possesses the calcium depletion effect^26^. Alvespimycin is reported to have lower toxicity than its lead compounds, geldanamycin and tanespimycin^24^. These are consistent with the above consideration based on the P14 factor score. Notably, all four Hsp90 inhibitors are located in quite near positions by clustering analysis^27^, which supports the utility of OLSA in understanding Hsp90 inhibitor characteristics (Fig. 5c).

#### Decomposition of topoisomerase-inhibitor effect

Topoisomerase inhibitors have been employed as anti-cancer drugs and are highly active against many types of neoplastic diseases^28^. However, the anti-cancer compounds, particularly anthracyclines among them, often exhibit cardiotoxicity, which restricts the application of that type of anti-neoplastic agent^29^.

The OLSA results of anthracyclines (doxorubicin, daunomycin, and mitoxantrone) revealed that the P5, P15, P16, and P17 factor scores were commonly high in topoisomerase inhibitors including non-drug compounds and the P17 factor stood out among them (Supplementary Fig. S6). In addition to topoisomerase inhibitors, GW-8510 (a CDK2 inhibitor) and staurosporine (a multiple kinase inhibitor) exhibited high scores in the P17 factor. Therefore, the P17 factor is estimated to be one of the main effects of topoisomerase inhibitors and includes G1/S arrest^22,30,31^. Indeed, H-7 (a multiple kinase inhibitor with topoisomerase inhibition activity), GW-8510, and alsterpaullone (a multiple CDK inhibitor) exhibited high Spearman’s correlation coefficients with the P17 factor in the test data set (Supplementary Fig. S6).

**Figure 6:**
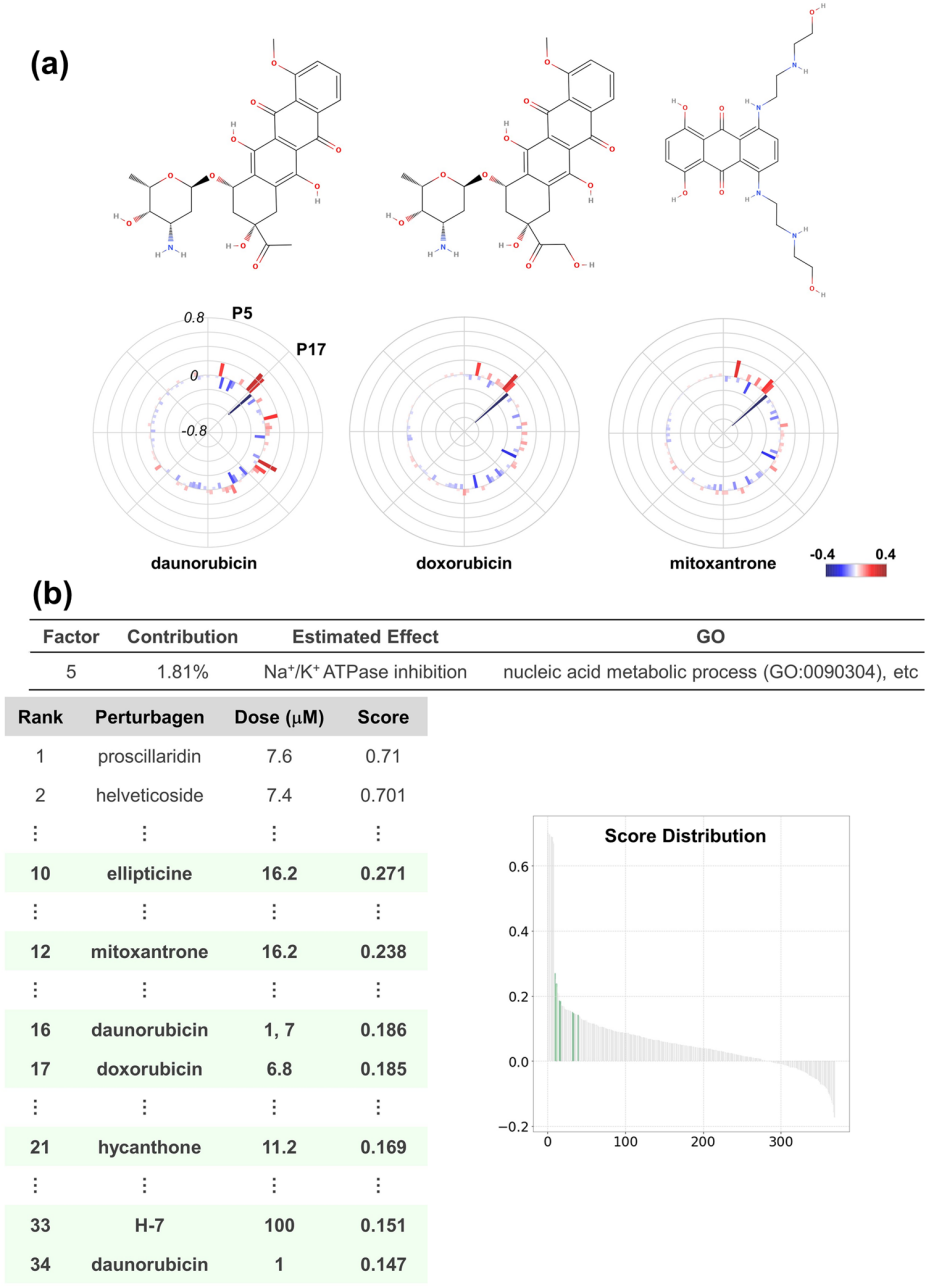
Decomposition of topoisomerase-inhibitor effect. (**a**) Structures and polar charts of response scores of topoisomerase inhibitors: daunorubicin, doxorubicin, and mitoxantrone. For daunorubicin, 7 μM-dose data were employed considering the higher effect on the transcriptional network than that of 1 μM. Structures were obtained from MolView (http://molview.org/). Response scores are plotted as a bar chart in polar co-ordinates with heatmap. (**b**) Analysis of the P5 factor. P5 factor scores of all compounds are arranged in descending order and plotted on the “Score Distribution” graph. Green in the graph indicates topoisomerase inhibitors. The rank, name, dose, and score are shown.

The P15 and P16 factor constituting genes exhibited significant enrichment of GO. However, we were not able to detect the commonality of the compounds in those factors other than topoisomerase inhibitors, and not able clearly to determine the cellular responses of the factors although P15 constituting genes seem to be associated with mitochondria (Supplementary Fig. S6). By contrast, the P5 factor was annotated with Na^+^/K^+^ ATPase inhibition in Fig. 2. Several studies reported Na^+^/K^+^ ATPase inhibition by doxorubicin^32^. Notably, one of the mechanisms explaining the cardiotoxicity induced with topoisomerase inhibitors is the inhibition of Na^+^/K^+^ ATPase^33^. This hypothesis is consistent with the relatively high scores for the P5 factor that topoisomerase inhibitors exhibited (Fig. 6b).

These results indicate that it is possible to decompose the effect of a drug with OLSA and imply that OLSA can detect not only the main effect of a drug, but also other effects that may be the cause of toxicity.

#### Identification of autophagy regulators

**Figure 7:**
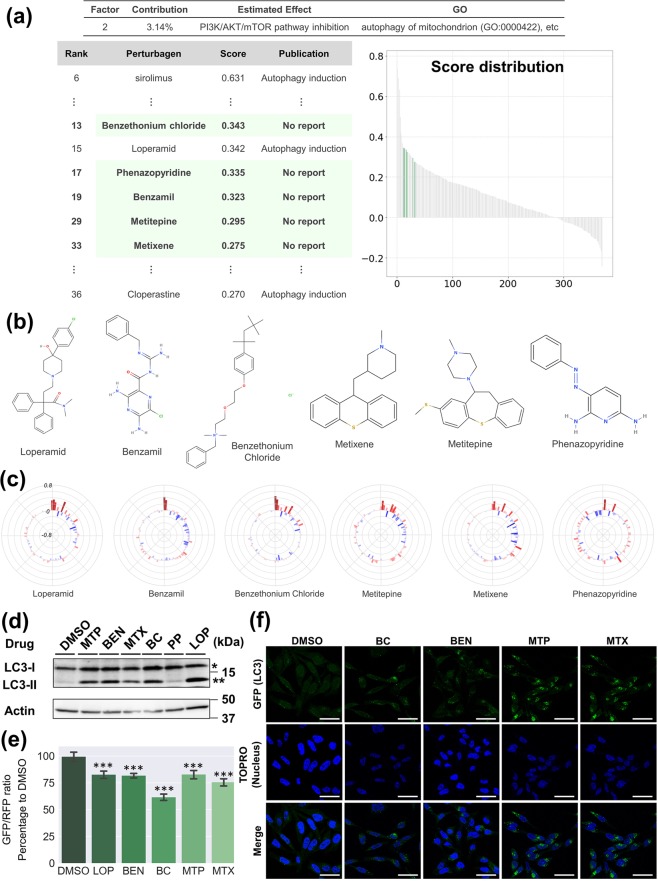
Identification of autophagy regulators. (**a**) Analysis of the P2 factor. P2 factor scores of all compounds are arranged in descending order and plotted on the “Score Distribution” graph. Green in the graph indicates the compounds with a high P2 score, but without reports about autophagy. The rank, name, dose, and score are shown. (**b**) Structures of the compounds tested in this study. Structures were obtained from MolView (http://molview.org/). (**c**) Polar charts of response scores of the compounds tested. Response scores are plotted as a bar chart in polar co-ordinates with heatmap. (**d**) Western blotting analysis of HeLa cells treated with the compounds tested. HeLa cells were treated with the compounds tested at the indicated concentration for 24 h. The whole-cell lysate was analysed by western blotting using anti-LC3 antibody. *LC3-I, **LC3-II. Full-length blots are presented in Supplementary Fig. S7d. (**e**) Autophagy flux evaluation of GFP-LC3-RFP-LC3ΔG-HeLa cells treated with the compounds tested. HeLa cells expressing GFP-LC3-RFP-LC3ΔG were treated with the compounds tested using the method described in D. GFP and RFP signals were quantified with a Tecan Infinite M200 plate reader and the GFP/RFP ratio was calculated. Each bar represents the mean ± SE, *n* = 6. Significance test was conducted with the Turkey–Kramer method and only significant differences between DMSO and the tested compounds are shown: ***P < 0.001. (**f**) Imaging analysis of GFP-LC3-RFP-LC3ΔG-HeLa cells treated with the compounds tested. HeLa cells expressing GFP-LC3-RFP-LC3ΔG were treated with the compounds tested using the method described in D, fixed with 4% paraformaldehyde, stained with TO-Pro-3 iodide, and the fluorescence signals were detected with a TCS SP5 confocal microscope. Green signals indicate GFP (LC3) and blue signals the TO-Pro-3 iodide (nucleus). Scale bars correspond to 50 μm. In (**d**–**f**), a representative result of at least two independent experiments is shown.

Finally, we explored the possibility of OLSA for drug repositioning. The analysis of CMap-derived data suggested that the P2 factor includes a basic effect responding to PI3K/AKT/mTOR signalling inhibition (Supplementary Fig. S7). The mammalian target of rapamycin complex (mTORC) I is a critical regulator of autophagy and its inhibition affects essential cellular phenomena^34^. We noticed that many compounds in the top 10% (37) of the list sorted by P2 factor scores were reported to be associated with autophagy (Supplementary Fig. S7). By contrast, there was no information regarding any autophagy relationship in 8 compounds on the list: 0297317-0002B, thonzonium bromide, benzethonium chloride (BC), methylbenzethonium chloride, phenazopyridine (PP), benzamil (BEN), methiothepin (MTP), and metixene (MTX). Therefore, we hypothesized that those compounds were related to autophagy regulation and tested the hypothesis. Among them, 0297417-0002B, thonzonium bromide, and methylbenzethonium chloride were excluded from the test compounds because the former was not easily available and the latter two were the same type of cationic detergent as BC. The remaining compounds, BC, BEN, MTP, MEX, and PP were subjected to *in vitro* analysis (Fig. 7a–c). Interestingly, those 5 compounds were not clustered with typical autophagy regulators such as sirolimus and wortmannin by clustering analysis (Supplementary Fig. S7). HeLa cells, a human cervical cancer-derived cell line, were treated with the tested compounds and loperamide (LOP), as a positive control^35^ and the conversion of LC3-I to LC3-II was analysed with western blotting. We employed LOP because it exhibited similar scores to those tested compounds and was reported as an autophagy inducer in more than two independent reports^35,36^. The conversion, one of the indicators of autophagy induction^37^, was clearly increased by 4 of 5 compounds (BC, BEN, MTP, and MTX), while PP had almost no effect (Fig. 7d).

GFP-LC3-RFP-LC3ΔG, an autophagic flux probe, is cleaved into equimolar amounts of GFP-LC3 and RFP-LC3ΔG by endogenous ATG4. The former is degraded in lysosome via autophagosome while the latter remains in the cytosol and works as an internal control. Thus, calculation of the GFP/RFP fluorescence ratio enables the precise estimation of autophagic flux^38^. GFP-LC3-RFP-LC3ΔG-expressing HeLa cells were treated with BC, BEN, MTP, and MTX and the GFP/RFP fluorescence ratio was measured. All 4 compounds reduced the ratio, which suggests they are autophagy inducers (Fig. 7e). GFP-LC3 puncta were clearly observed under the same condition (Fig. 7f). All of the results indicate that BC, BEN, MTP, and MTX are autophagy inducers. Four of five compounds predicted to be autophagy regulators by OLSA actually induced autophagy, which supports one of the utilities of this method in drug repositioning.

## Discussion

Drug discovery with drug repositioning has been a successful approach^39–41^. The approach mostly depends on serendipity in the beginning, but the various methodologies based on scientific evidence have been established, which contribute to the development of the approach^42–44^. The success of drug repositioning implies that the effect of a drug is complex and comprises multiple basic components. Therefore, here we attempted to separate the complex effect of a drug into basic effects to understand the pharmacological properties of drugs. To achieve such separation, we focused on profile data analysis. An important characteristic of omics is the comprehensive conversion of the biological information of a sample into numeric values, which enables mathematical approaches to the analysis of biological samples. However, in general, raw omics data are multiple variables, complex, and often difficult to comprehend because of the curse of dimensionality^45^. Analytical methods setting a new layer that appropriately contracts the degrees of freedom are indispensable for extracting information from the data and many methods have been devised^46,47^. The factors generated with OLSA may be such new indicators constituting a novel layer and may compress biological information.

We should estimate the biological meanings of the new indicators cautiously, because contracting profile data with OLSA is conducted in an unsupervised manner and interpretation depends on the analysts. We have attempted to estimate meanings using two approaches: the first is to analyse the variables mainly constituting a factor and another is to utilize the similarity of the high-ranked samples in the list sorted by the response-score ranking of the focusing factor. When transcriptome profile data were subjected to OLSA, the variables were genes. To obtain insight into whether a factor has consistent biological meanings or not, we used statistical significance in a GO analysis of the main genes constituting the focused factor and evaluated the correspondence to the existing bodies of knowledge as a requirement. Interestingly, the ratio of the factors with significant enrichment of GO varied between the data sets and SEGR was 0.551, 0.724, and 0.333 in CMap, HepG2, and BMDM response-profile matrices, respectively (Figs 2b, 3b, and Supplementary Fig. S4). An explanation of the differences is the possibility that the contribution ratio of biologically relevant factors is different between the data sets. In the data sets with relatively low SEGR (CMap and BMDM set), the factors annotated with GO tended to contribute highly to the total deviation and the significance of the enrichment was supported by the results of Fisher’s exact tests (Supplementary Fig. S8). Moreover, in some of the factors with a high contribution ratio, but without GO annotation, for instance the P26 and P35 factors, structurally similar compounds dominate the top of the score ranking, which implies the association with some cellular responses (Supplementary Fig. S2). OLSA is a matrix-decomposition method based on FA; therefore, a factor with a high contribution ratio means that the factor includes a response with large variance. As GO is a classification method based on existing knowledge, the relationship between factor contribution and annotation with GO in our results is consistent with our daily experience: a phenomenon with strong phenotype is easily detected, while a weak phenotype is often missed.

The factors can be classified into 4 types based on where the contracted deviation is derived from: (1) a biological response characterized well, (2) a biological response not characterized or identified, (3) biological responses that are non-linear and cannot be separated, and (4) noise. The former two are biologically relevant and, in particular, analysing the second type may lead to new biological findings. However, it is difficult to distinguish between noise, uncharacterized, and the unseparated factors. Mathematical considerations focusing on the contribution ratio may be useful for setting the criteria to distinguish between the biologically relevant factors and not relevant ones, although we have applied the generally used threshold in PCA and analysed the cumulative contribution ratio of factors in the present study^14^. The relationship between factor contribution and biological relevance is an important point of OLSA and its analysis is an essential future task.

We consider that OLSA is a research tool for assessing the purity of the effects of a candidate compound group, thereby facilitating the lead optimization process for drug discovery^48,49^. OLSA provides scores of the common factors in the group and compound-specific factors among cellular responses to the candidate compounds. It may be possible to prioritize the candidates according to the purity of their effects based on the scores of common effects. Selecting the candidates with high purity is expected to be useful for avoiding toxicity specific to that particular candidate, nevertheless, it is often difficult to discriminate such response. The common factors are expected to include the effects based on the mode of action of the candidates—so-called “class effects”—leading to a deeper understanding of their structure–activity relationships and the rational design of potential drugs. Thus, OLSA has the potential to be useful for determining the biochemical assays necessary for the next step in the lead optimization process of drug discovery.

In the present study, we tested whether the factors obtained by OLSA could be used for drug repositioning, focusing on the P2 factor in CMap data; a factor expected to include PI3K/AKT/mTOR inhibition and to be associated with autophagy via mTORC1 modulation. Western blotting and the following analysis revealed that 4 of 5 tested compounds actually induced autophagy, while this prediction was not achieved by conventional clustering analysis (Supplementary Fig. S7). The results support the potency of the drug effect separation strategy. However, we should carefully discuss why PP did not induce autophagy, contrary to the prediction. One of the reasons is considered to be the discrepancy between the wanted cellular response for repositioning and the estimated response, particularly in cases where the cellular response is regulated by several factors. For instance, LY294002 and wortmannin are listed as P2V high score compounds and reported to inhibit mTORC1 activity as predicted, but do not induce autophagy because they are pan-inhibitors of PI3K^50,51^. The PI3K family is divided into three classes^52^. Class I PI3K activates mTOR via PI3K/AKT/mTOR signalling and reduces autophagy, while Class III PI3K increases phagophore formation and promotes autophagy. Because of this two-sided effect, pan-PI3K inhibitors do inhibit mTORC1, but do not induce autophagy^50,51^. We confirmed that PP decreased phosphorylation of S6K, an mTORC1 substrate and an mTORC1 activation marker, which indicates that PP does have an mTORC1 inhibitory effect as predicted (Supplementary Fig. S7). Thus, OLSA actually contributed to the understanding of a perturbagen effect by separating it, whereas the wanted cellular response for drug repositioning does not always correspond directly to the estimated response. It may be helpful for a drug repositioning strategy using OLSA to consider the relationship between the factors using topological techniques such as graphical modelling^53^.

Here we highlight the potential of OLSA to dissect a drug effect and extract its basic components by analysing transcriptome data. Because OLSA does not require existing knowledge or biological meaning of the variables in profiles, this method can be applied to the data such as the spots in two-dimensional electrophoresis and features in phenotyping screening^54^. Moreover, as shown in Fig. 4, perturbagens are not limited to drugs and any profile change from the control (response profile) can be subjected to OLSA. These attributes suggest wide application of this method. We expect that OLSA will contribute to drug repositioning, lead optimization, and other approaches in drug discovery.

## Methods

### Data pre-processing for OLSA input

The expression data matrix was prepared as variables in rows and samples in columns. Here, we describe the procedures considering transcriptome data, although the methodology can be applied to other types of omics data. Data from each sample were converted into a non-parametric rank-ordered list of all genes in the transcriptome data based on the expression values (expression rank matrix). To obtain differential expression values to the controls, we employed a robust *z*-scoring procedure and the differential expression value gene *x* to the control in the *i*th sample was computed as:

$${z}_{i}=\frac{{x}_{i}\,-median(Y)}{NIQR},$$

where *x* and *Y* are the vectors of gene *x* ranks across all samples and the control samples in the expression rank matrix, respectively, and *NIQR* is the normalized interquartile range of the control sample values. We define a response-profile matrix *D* as:

$$D=({z}_{1}\,{z}_{2}\,\cdots \,{z}_{N}),$$

where *z*_*i*_ is the column vector consisting of z-scores of the *i*th sample and *N* is the number of the samples in the data set.

### OLSA

OLSA consists of the following procedures. The concept and workflow are shown in Supplementary Fig. S1.

#### Data selection for mirror data set

To exclude the samples that are considered to lose reversibility, the outlier samples with extremely large differential expression values are removed from the response-profile matrix. The L2-norm of *i*th sample is calculated as:

$${l}_{i}={(\sum _{k}^{n}{{z}_{ik}}^{2})}^{\frac{1}{2}},$$

where *z*_*ik*_ is a robust z score of genes of *i*th sample and *n* is the number of genes in the sample. L2-norms of all samples are subjected to the Smirnov–Grubbs test, and response profiles without an extremely large L2-norm are selected to prepare the mirror data set. Pre-mirror data matrix *P* is defined as:

$$P=({z}_{1}\,{z}_{2}\,\cdots \,{z}_{m}),$$

where *z*_*i*_ is the column vector consisting of z-scores of the *i*th sample among the selected profiles and *m* is the number of the selected samples. We call the diagonal matrix consisting of L2-norm “total strength” and the matrix *T* is defined as:

$$T=(\begin{array}{ccc}{l}_{1} & \cdots  & 0\\ \vdots  & \ddots  & \vdots \\ 0 & \cdots  & {l}_{N}\end{array}).$$

#### Normalization

Each response profile in the response-profile matrix *D* and the selected matrix *P* is normalized by corresponding L2-norm and the normalized response-profile matrix is defined as *D*′ and *P*′, respectively. This procedure makes it possible to compare each variable between samples and to analyse the relationship between samples in a manner independent of the strength of the stimulation.

#### Mirror data preparation

A point-symmetric data set *M* to the selected normalized data set *P*′ is generated as:

$$M=-\,P^{\prime} .$$

Then, we concatenate the normalized data set *D*′ and this mirror data set *M* in a row, which makes the centroid of this combined data set zero. We define the concatenated matrix as *DM*.

#### Principal component analysis

The concatenated data set *DM* is subjected to principal component analysis with the Scikit-learn library of Python 3 to contract the gene expression changes with their co-ordination, which generates a matrix consisting of the obtained components, *C*.

#### Varimax rotation

To reduce the genes contributing to each contracting vector retaining the orthogonality of the vectors, varimax rotation is applied to *C*. We describe the python code for this process as “rotateV” in Supplementary Data. In this work, the vectors are sorted by contribution ratio, and those from the top to the vector whose cumulative contribution ratio exceeds 0.8 (CMap and Magkoufopoulou’s data) or 0.9 (Raza’s data) are subjected to varimax rotation considering calculation time. Here, “contribution ratio” and “cumulative contribution” indicate the ratio of the variance of a factor to the total variance and the sum of variances from the top to the focusing factor in the contribution ratio list sorted in descending order, respectively. We define the rotated vectors as “response vectors” (“factors”) and call a matrix consisting of response vectors the “response-vector matrix”, *R*.

#### Generation of the response-score matrix

Employing the response-vector matrix *R*, the scores of the samples for each factor are calculated as inner products. We label a matrix consisting of the scores the “response-score matrix” *S* as:

$$S=R\cdot D^{\prime} .$$

### GO analysis of genes mainly constituting the response vector

Genes constituting a response vector were sorted by each corresponding absolute value. The top 1% of genes were subjected to GO (biological process) analysis using the Enrichment analysis of Gene Ontology Consortium (http://www.geneontology.org/). The gene lists subjected to GO analysis and the results are provided in each corresponding Supplementary Data as a book named “Gene_List_forGO” and “GO_Result”, respectively. The obtained *p*-values were processed using the Benjamini–Hochberg method for multiple-testing corrections among factors (α < 0.05). The results are provided in each corresponding Supplementary Data as a book named “Summary.”

### Validation of biological meaning of response vector

We validated the estimated biological response of a response vector annotated with GO, the treatment similarity, or the results of a literature survey by investigating Spearman’s correlations (described as rho or ρ) between the factor constituents and the samples in another data set. First, the contribution of each gene constituting a response vector was calculated as the ratio of the square of each gene value to the summation of those values and then sorted by the ratio. Genes from the top to the gene whose cumulative contribution ratio exceeded 0.9 in the list were selected and employed as the signature representing the factor to calculate Spearman’s correlation.

### Materials

Benzethonium chloride (025–11662), loperamide hydrochloride (129–05721), and phenazopyridine hydrochloride (162–14441) were purchased from Wako Pure Chemical Industries (Osaka, Japan). Methiothepin hydrochloride (sc-253005) and anti-β-Actin (sc-47778) were purchased from Santa Cruz Biotechnology (Dallas, TX). Benzamil (3380) was purchased from Tocris Bioscience (Bristol, UK). Metixene hydrochloride (M1808000) and bafilomycin A1 from *Streptomyces griseus* (B1793) were purchased from Merck (Darmstadt, Germany). Rabbit anti-p70 S6 kinase (9202) and mouse anti-phospho-p70 S6 kinase (9206) were purchased from Cell Signaling Technology (Beverly, MA). Rabbit anti-LC3 (PM036) was purchased from Medical and Biological Laboratories (Nagoya, Japan). All other chemicals were of analytical grade.

### Cell culture

GFP-LC3-RFP-LC3ΔG-expressing HeLa cells were cultured in Dulbecco’s modified Eagle’s medium (DMEM) (D6546, Merck) supplemented with 10% fetal bovine serum (FBS) and 2 mM l-glutamine (G7513, Merck). HeLa cells (CCL-2, ATCC) were cultured in DMEM (10313–021, Life Technologies, Carlsbad, CA) with 10% FBS and 1% MEM non-essential amino acids (11140–050, Life Technologies). All cells were maintained at 37 °C under 5% CO_2_.

### Western blotting analysis

Western blotting analysis was conducted as previously described^55^. Specimens were separated with sodium dodecyl sulfate polyacrylamide gel electrophoresis on a 13.5% polyacrylamide gel with a 3.75% stacking gel at 140 V for 90 min. The molecular weight was determined using Precision Plus Protein Standards (1610373, Bio-Rad, Richmond, CA). Proteins were transferred electrophoretically to a poly(vinylidene difluoride) (PVDF) membrane (Pall, NY) using a blotter (Bio-Rad) at 100 V for 60 min. Non-specific binding sites on the membrane were blocked with PVDF Blocking Reagent for Can Get Signal (Toyobo, Osaka, Japan) at room temperature for 60 min. After blocking, the PVDF membrane was incubated with primary antibodies diluted with Can Get Signal solution 1 (Toyobo) at 4 °C for 24 h. Primary antibodies were used in the following conditions: anti-β-actin (1/2,000), anti-p70 S6 kinase (1/2,000), anti-phospho-p70 S6 kinase (1/2,000), and anti-LC3 (1/2,000). After the reaction with primary antibodies, the membrane was incubated with horseradish peroxidase-conjugated anti-rabbit or anti-mouse IgG antibody (Amersham Biosciences, Piscataway, NJ) diluted to 1/10,000 in Tris-buffered saline containing 0.05% Tween 20 at room temperature for 60 min. Immunoreactivity was detected with a Fusion Solo S (Vilber Lourmat, Marne-la-Vallée, France) and Westar ETA C Ultra 2.0 (Cyanagen, Bologna, Italy). The band intensity indicating each protein was quantified by Multi Gauge software (Fujifilm, Tokyo, Japan).

### Evaluation of autophagic flux with GFP-LC3-RFP-LC3ΔG-expressing HeLa cells

Autophagic flux after drug treatment was determined essentially as described previously^35^. GFP-LC3-RFP-LC3ΔG-expressing HeLa cells were seeded in black/clear bottom 96-well plates (353948, Corning, NY) at 1.5 × 10^4^ cells/well and maintained for 72 h. After drug treatment for 6 h, cells were washed with PBS (+), fixed with 4% paraformaldehyde solution (163–20145, Wako) for 10 min, and washed with PBS (+). Measurement of GFP and RFP fluorescence was performed using a microplate reader (Infinite M200 microplate reader; Tecan, Mannedorf, Switzerland) with excitation/emission at 480/510 nm and 580/610 nm, respectively.

### GFP-LC3 imaging

Fluorescence microscopy was conducted as described previously^56^. GFP-LC3-RFP-LC3ΔG-expressing HeLa cells were seeded on glass coverslips (Matsunami Glass, Osaka, Japan) in 12-well plates at 1.5 × 10^4^ cells/well and maintained for 72 h. After drug treatment for 24 h, cells were washed twice with PBS, fixed with 4% paraformaldehyde solution for 10 min, permeabilized with 0.1% saponin in PBS for 10 min, and blocked with 3% bovine serum albumin (BSA) in PBS for 30 min. After blocking, cells were incubated with TO-Pro-3 iodide (Life Technologies, Carlsbad, CA) diluted to 1/2,500 by 2% BSA in PBS for 60 min. Coverslips were mounted with H-1000 Vectashield mounting medium (Vector Laboratories, Burlingame, CA) and analysed with a TCS SP5 confocal microscope (Leica, Solms, Germany). Images were processed with LAS AF (Leica).

### Statistical analysis

Student’s two-tailed unpaired *t* test and one-way analysis of variance followed by Tukey’s *post hoc* multiple comparison test were used to identify significant differences among groups, where appropriate. The data were analysed using Prism software (GraphPad Software, La Jolla, CA) and Scikit-learn library of Python 3.

## Supplementary information

Supplementary Information

Supplementary Dataset For Figure 2

Supplementary Dataset For Figure 3

Supplementary Dataset For Figure 4

Supplementary Dataset For Figure 6

Supplementary Dataset For Figure 7

Supplementary Code

## Data Availability

The computer code produced in this study is available in Supplementary Code and in the following database: • OLSA python scripts: GitHub (https://github.com/tadahaya222/OLSApy).
