# Supplementary material for: Development of Orthogonal Linear Separation Analysis (OLSA) to Decompose Drug Effects into Basic Components
Source: Sci Rep. 2019 Feb 12;9:1824. doi: 10.1038/s41598-019-38528-4 (PMC6372619; doi:10.1038/s41598-019-38528-4)
Supplement: Supplementary file 1 — Supplementary Information [file 41598_2019_38528_MOESM1_ESM.docx]

**Title**

**Development of Orthogonal Linear Separation Analysis (OLSA) to Decompose Drug Effects into Basic Components**

**Tadahaya Mizuno*^, 1^, Setsuo Kinoshita^1, 2^, Takuya Ito^1^, Shotaro Maedera^1^, and Hiroyuki Kusuhara**^, 1^**

^1^Graduate School of Pharmaceutical Sciences, the University of Tokyo, Bunkyo-ku, Tokyo, 113-0033, Japan

^2^ProMedico Co., Ltd., Ota-ku, Tokyo, 143-0023, Japan

* Corresponding author: Tel: +81-3-5841-4771; E-mail: [tadahaya@mol.f.u-tokyo.ac.jp](mailto:tadahaya@mol.f.u-tokyo.ac.jp)

** Corresponding author: Tel: +81-3-5841-4770; E-mail: [kusuhara@mol.f.u-tokyo.ac.jp](mailto:kusuhara@mol.f.u-tokyo.ac.jp)

**Supplementary Methods**

**Fisher’s exact test between the factors with high contribution ratio and the ones with significant enrichment of GO**

A 2 × 2 contingency table for factors in CMap and BMDM data set are prepared with the threshold whether cumulative contribution ratio is less than 60% or not, and whether GO is significantly enriched or not. Fisher’s exact test is conducted with Scikit-learn library of Python 3.

**Supplementary Figure**


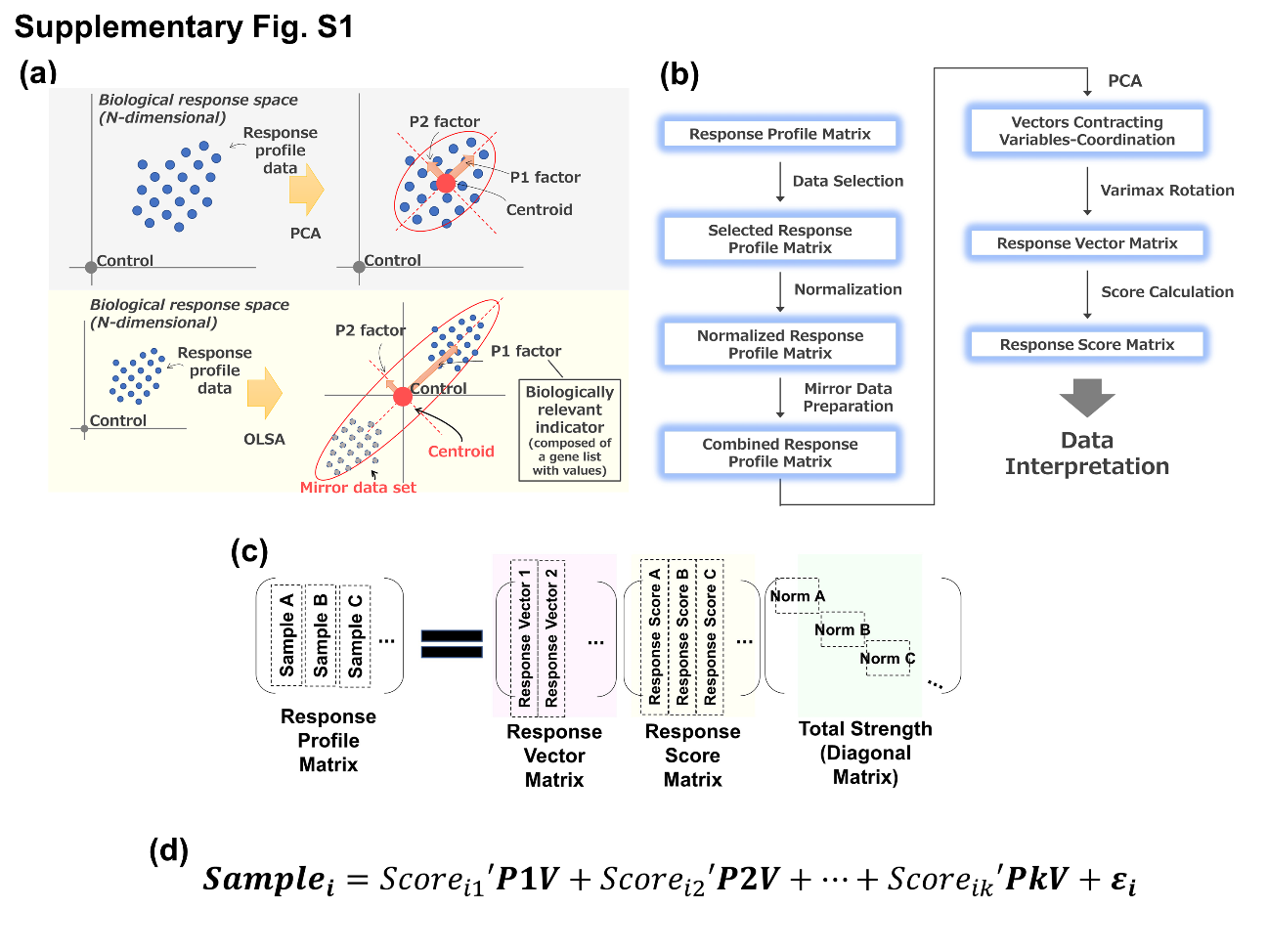


**Supplementary Fig. S1 The concept of orthogonal linear separation analysis of profile data**

a. Illustration of OLSA concept.

b. Workflow of orthogonal linear separation analysis (OLSA).

c. Definition and relation of response-profile matrix, response-vector matrix, response-score matrix, and total strength. A response-score matrix is decomposed into the inner product of a response-vector matrix, a response-score matrix, and a diagonal matrix of the L2-norm.

d. Description of a sample with a linear combination of the factors. When OLSA is applied to a response-profile data set with *p* variables in rows and *n* samples in columns, a sample is described in this way. $\boldsymbol{Sample}_{\boldsymbol{i}}$, the data of *i*th sample where *i* = 1, …, *n*; $\boldsymbol{Score}_{\boldsymbol{ik}}\boldsymbol{'}$, the response-score value of the *k*th response vector multiplied with the corresponding total strength;$\boldsymbol{PkV}$, the *k*th response vector; $\boldsymbol{\varepsilon}_{\boldsymbol{i}}$, unobserved stochastic error terms.


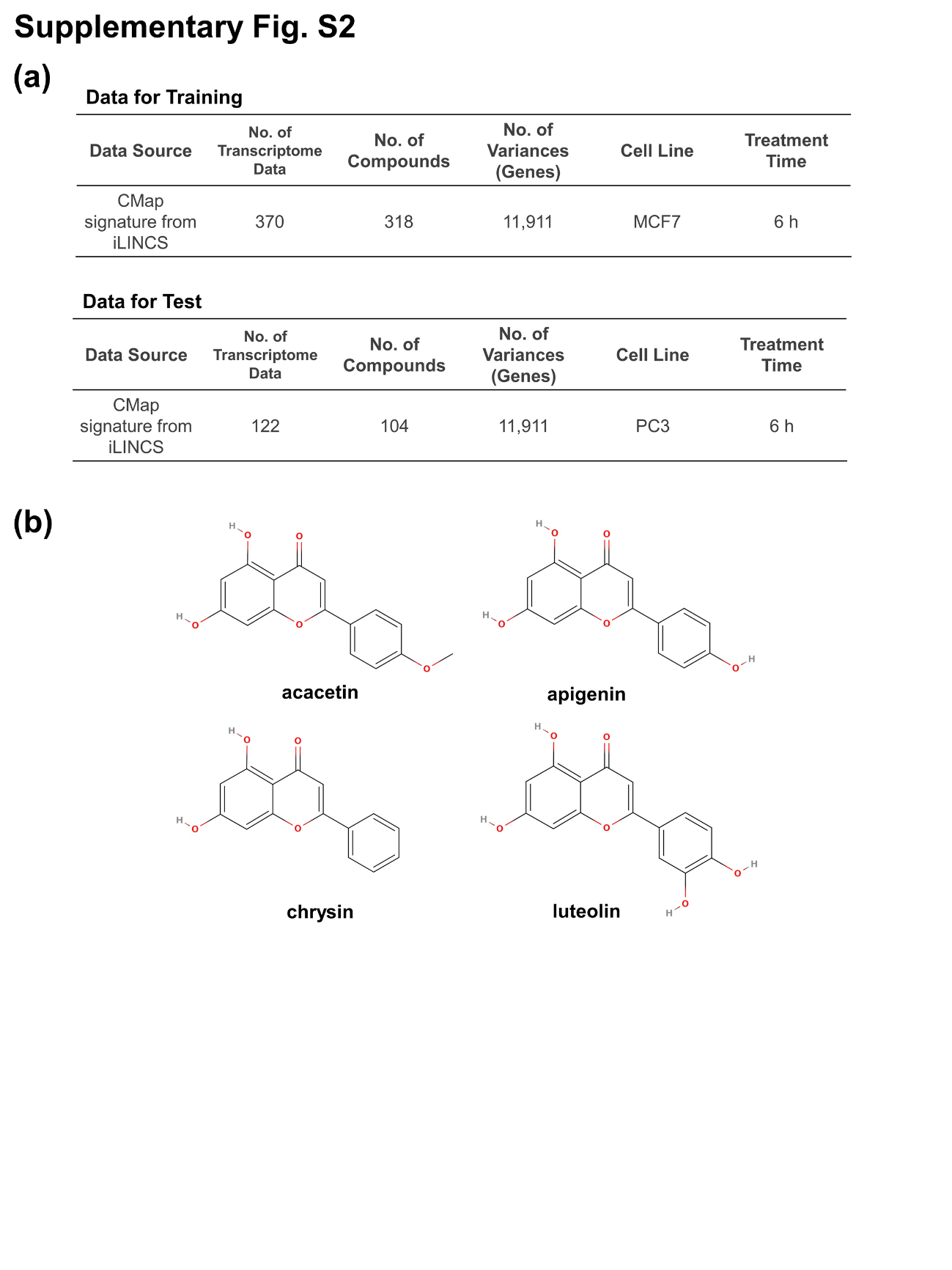


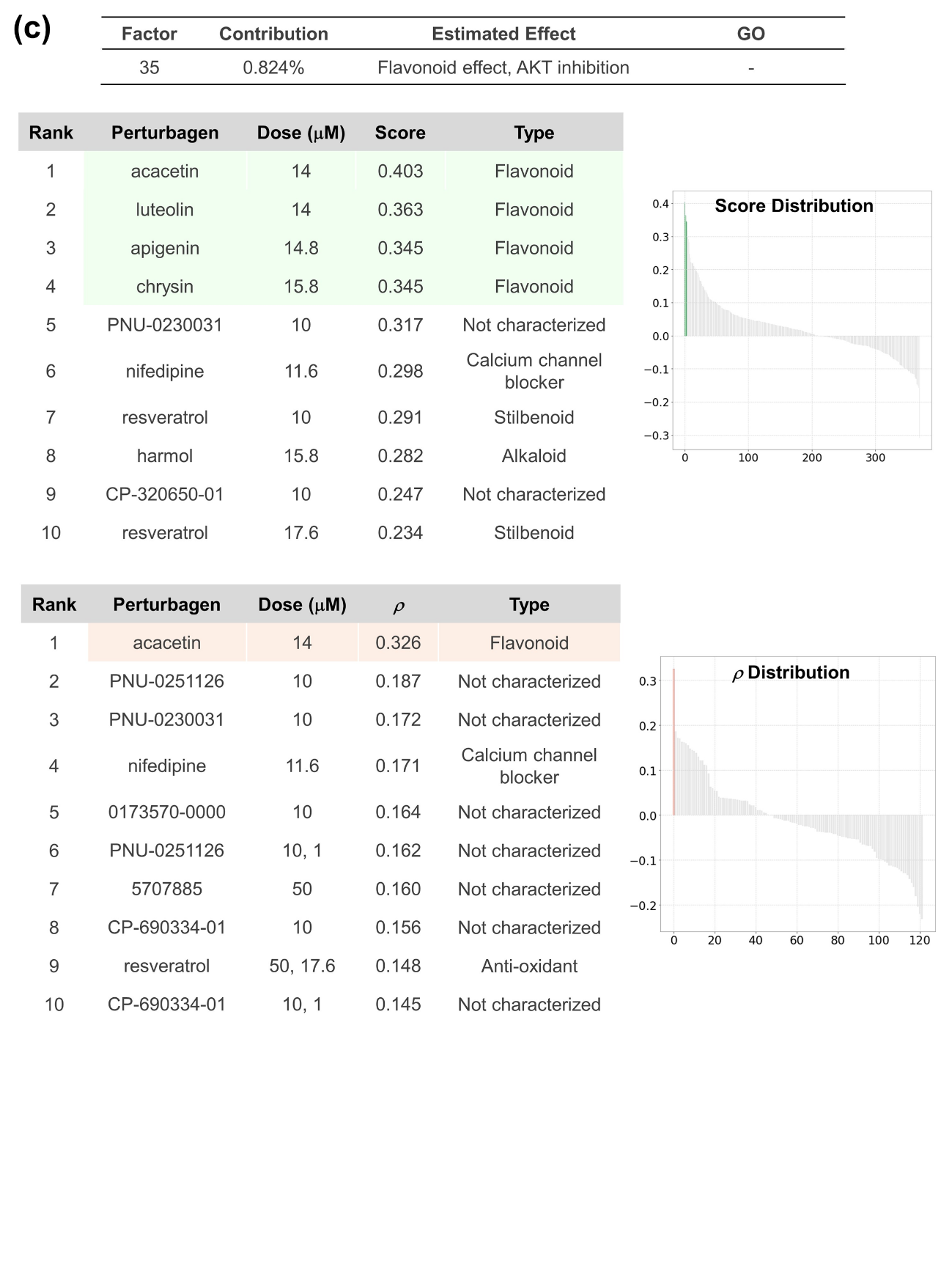


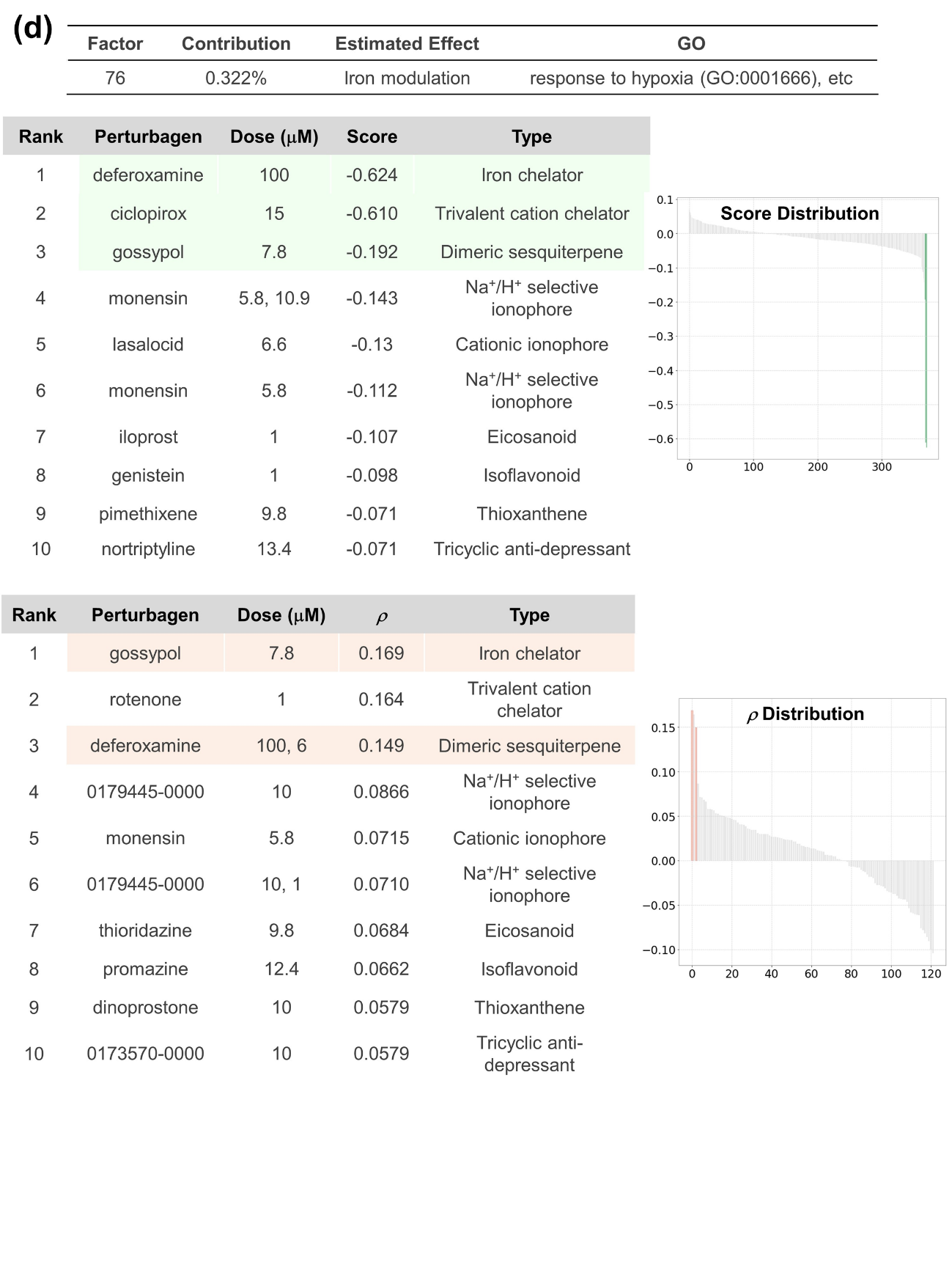


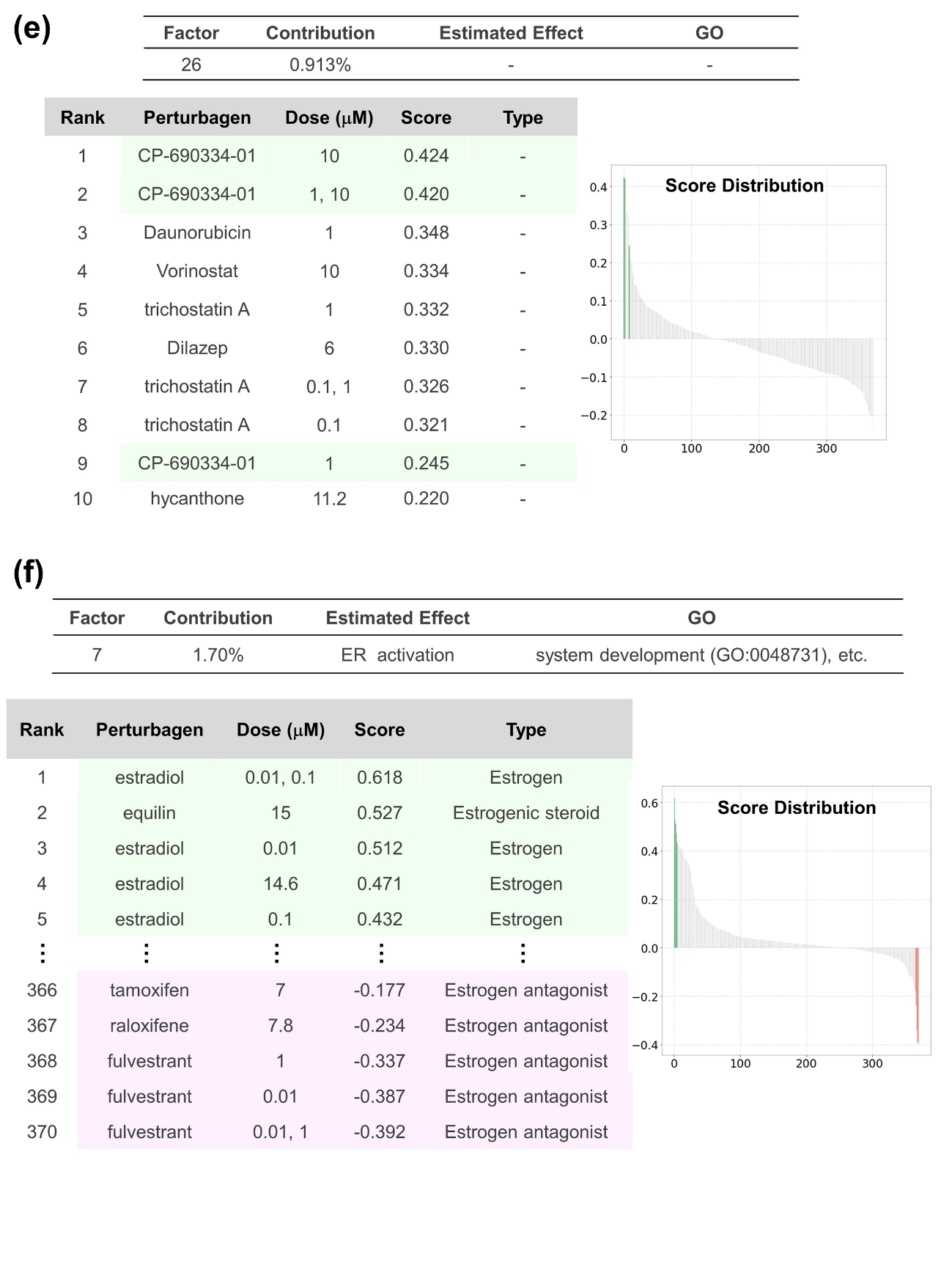


**Supplementary Fig. S2 Analysis of cellular responses in MCF7 cells treated with 370 perturbagens**

a. Data sets employed in this figure.

b. Structure of flavonoids in the data sets.

c. Analysis of the P35 factor using the method shown in Fig. 2c.

d. Analysis of the P76 factor using the method described in Fig. 2c.

e. Analysis of the P26 factor score using the method described in Fig. 2c.

f. Analysis of the P7 factor. Green and red in the graph indicate estrogens and antiestrogens, respectively. The rank, name, dose, and score of the top and last 5 compounds are shown.


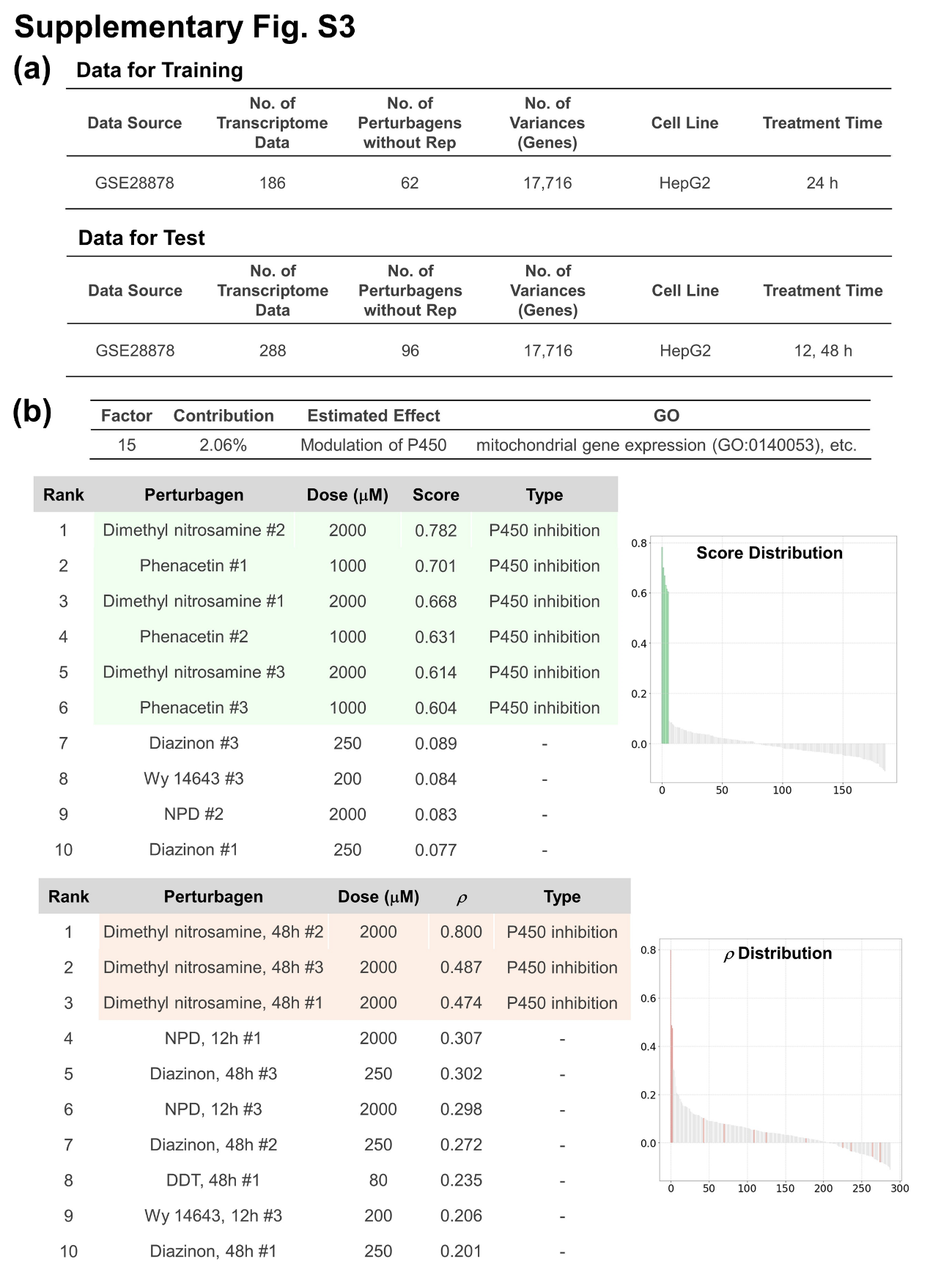


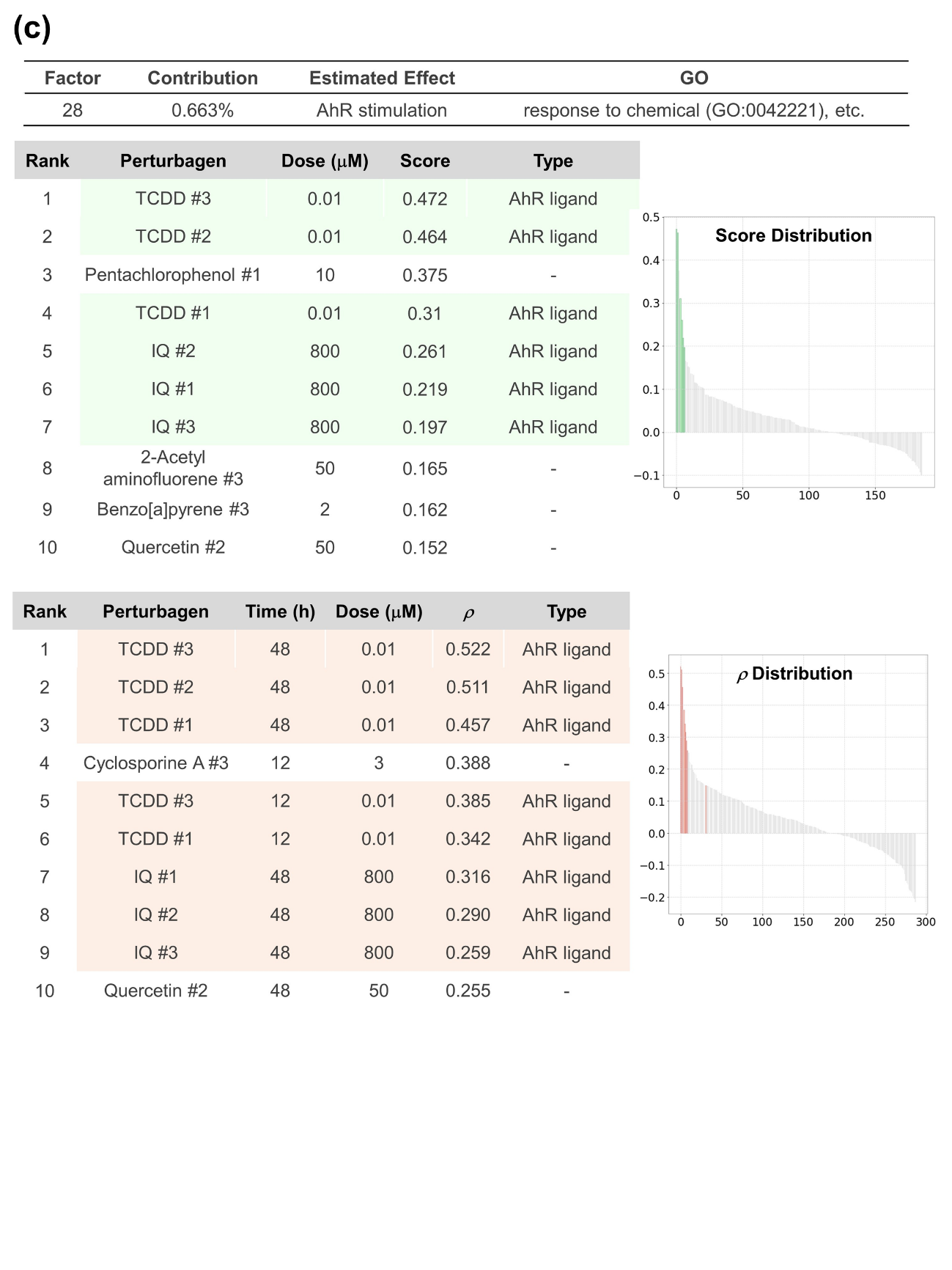


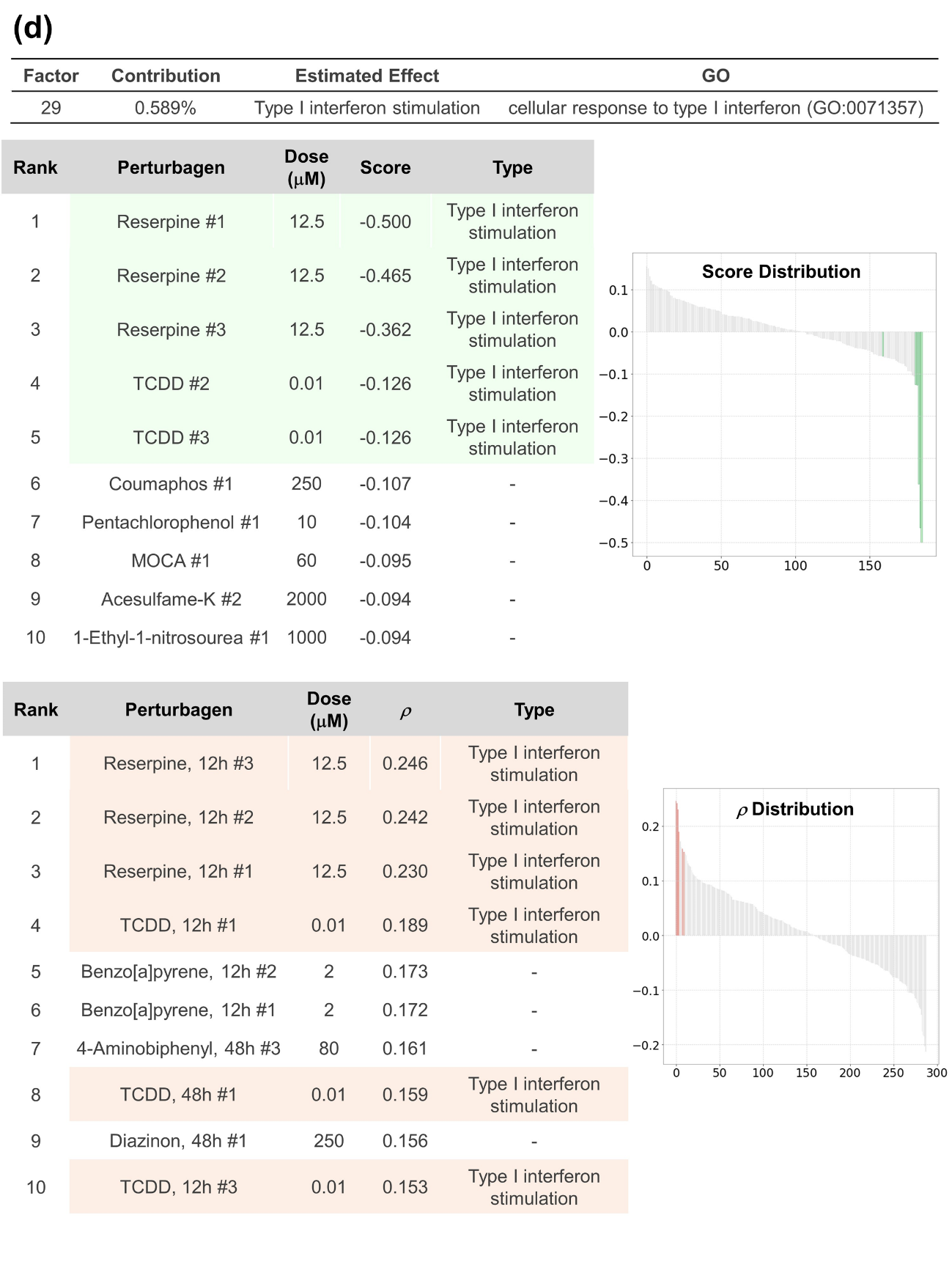


**Supplementary Fig. S3 Analysis of cellular responses in HepG2 cells treated with 62 genotoxic compounds**

a. Data sets employed in this figure.

b. Analysis of the P15 factor using the method described in Fig. 3c. Green or light salmon in the graph indicates dimethyl nitrosamine or phenacetin. NPD, 4-nitro-*o*-phenylenediamine.

c. Analysis of the P28 factor using the method described in Fig. 3c. Green or light salmon in the graph indicates TCDD or IQ, respectively. TCDD, 2,3,7,8-tetrachlorodibenzo-*p*-dioxin; IQ, 2-amino-3-methylimidazo[4,5-*f*]quinolone.

d. Analysis of the P29 factor using the method described in Fig. 3c. Green or light salmon in the graph indicates reserpine or TCDD. TCDD, 2,3,7,8-tetrachlorodibenzo-*p*-dioxin; MOCA, 4,4′-methylene-bis(2-chloroaniline).


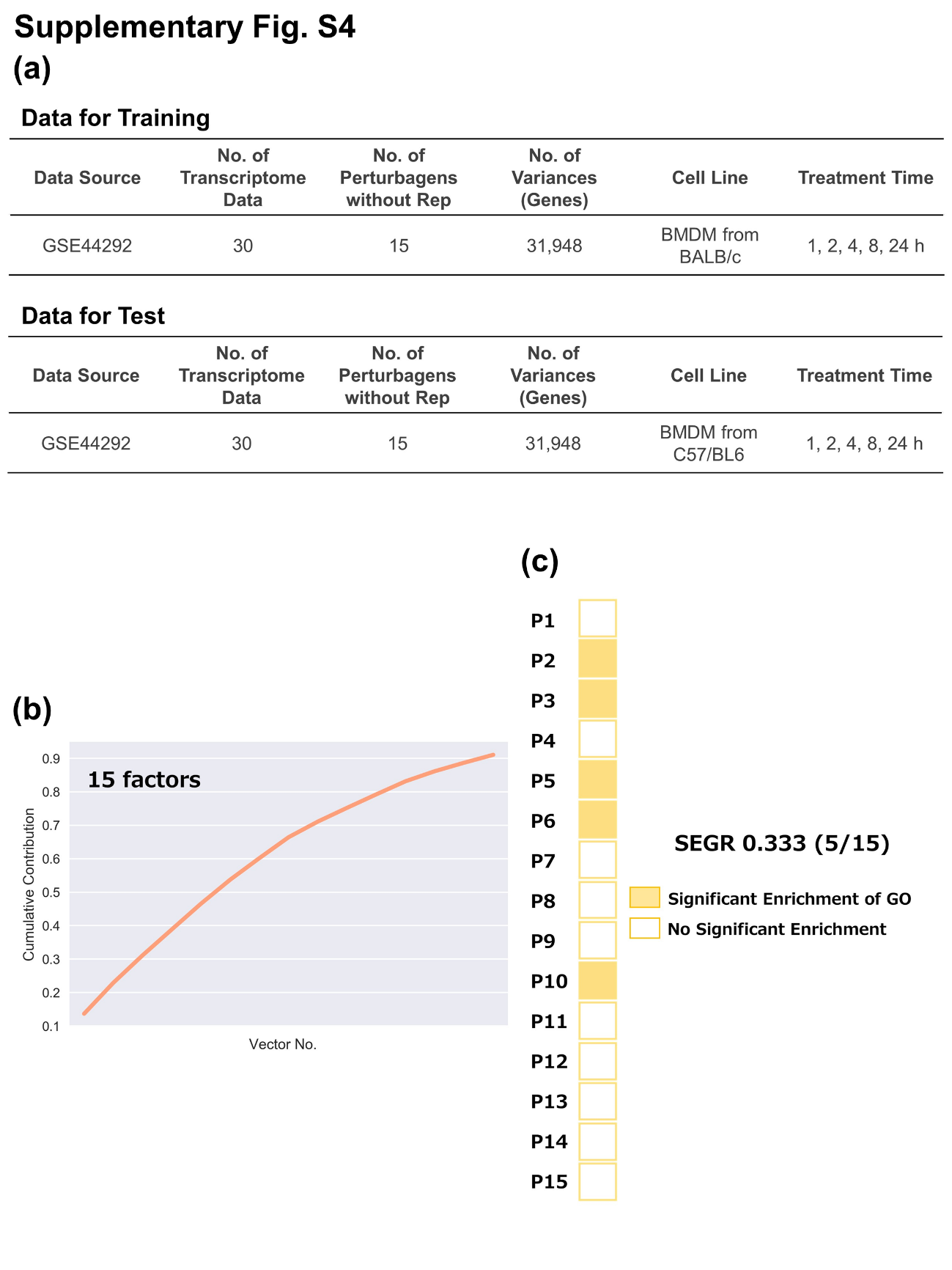


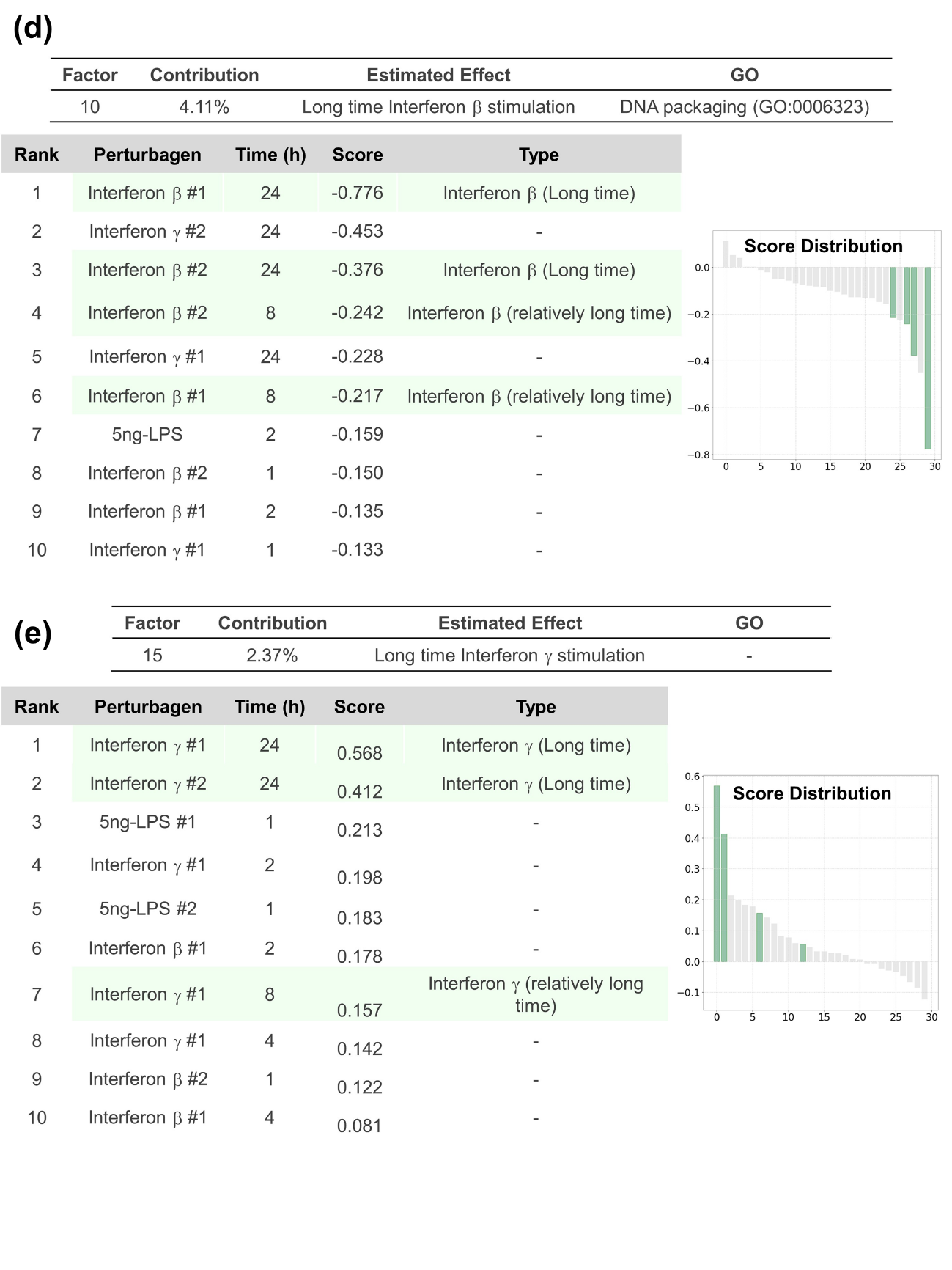


**Supplementary Fig. S4 Analysis of inflammatory responses in macrophages**

a. Data sets employed in this figure.

b. The cumulative contribution curve of the factors comprising the training data set. The contribution of each factor to the total deviation was calculated and arranged in descending order. The cumulative contribution was calculated from the top and plotted.

c. Plot of the factors whose main constituents exhibit significant enrichment of gene ontology. Genes constituting a response vector were sorted by the square of each value. The top 1% of genes were subjected to GO (biological process) analysis using Enrichment analysis of Gene Ontology Consortium. Factors annotated with significant enrichment of GO after multiple-testing corrections (Benjamini–Hochberg method, α < 0.05) were depicted in yellow-filled squares. SEGR, significant enrichment of GO.

d. Analysis of the P10 factor using the method shown in Fig. 4a. Green in the graph indicates 24- or 8-h interferon β treatment.

e. Analysis of the P15 factor using the method described in Fig. 4a. Green in the graph indicates 24- or 8-h interferon γ treatment.


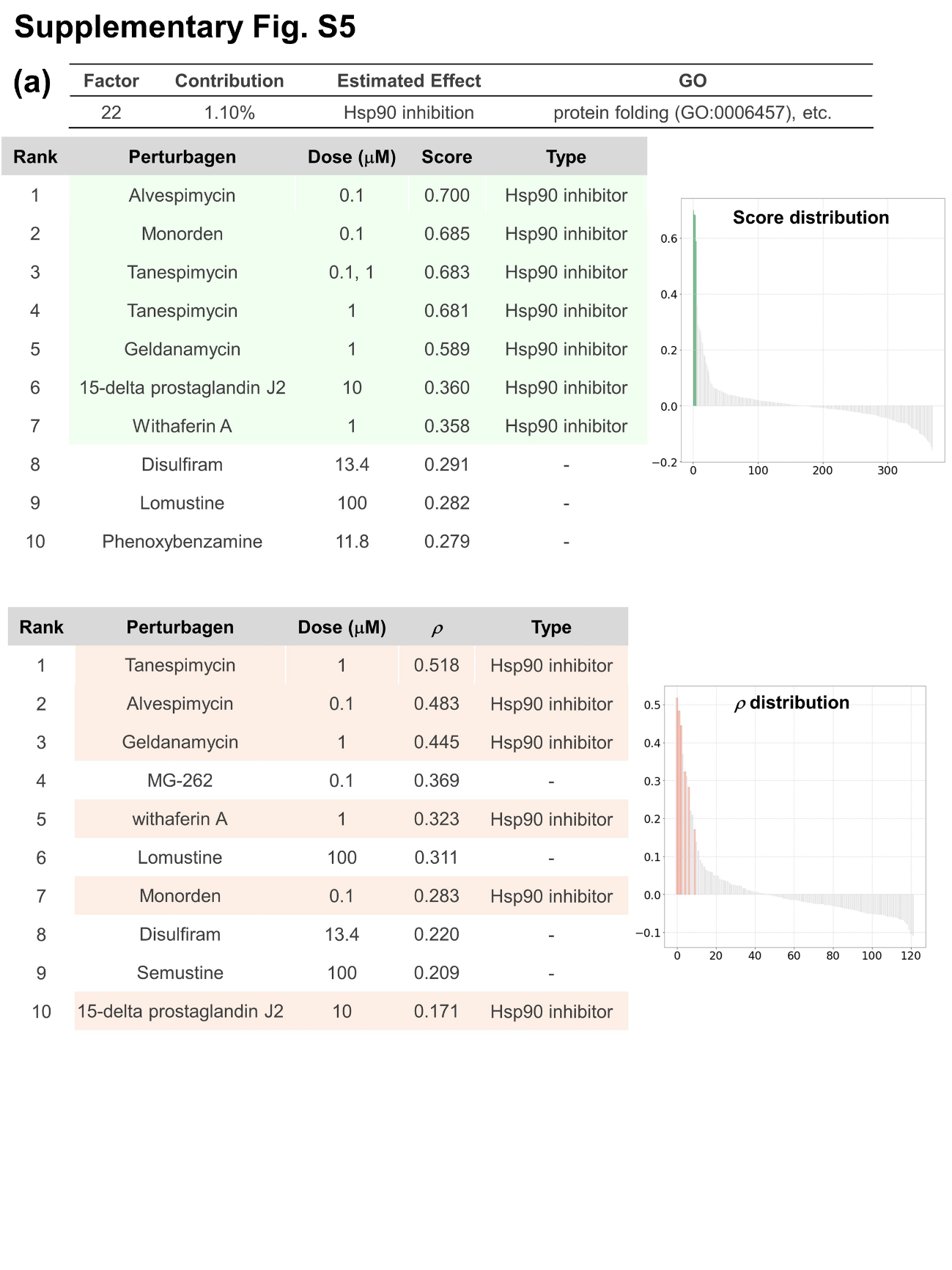


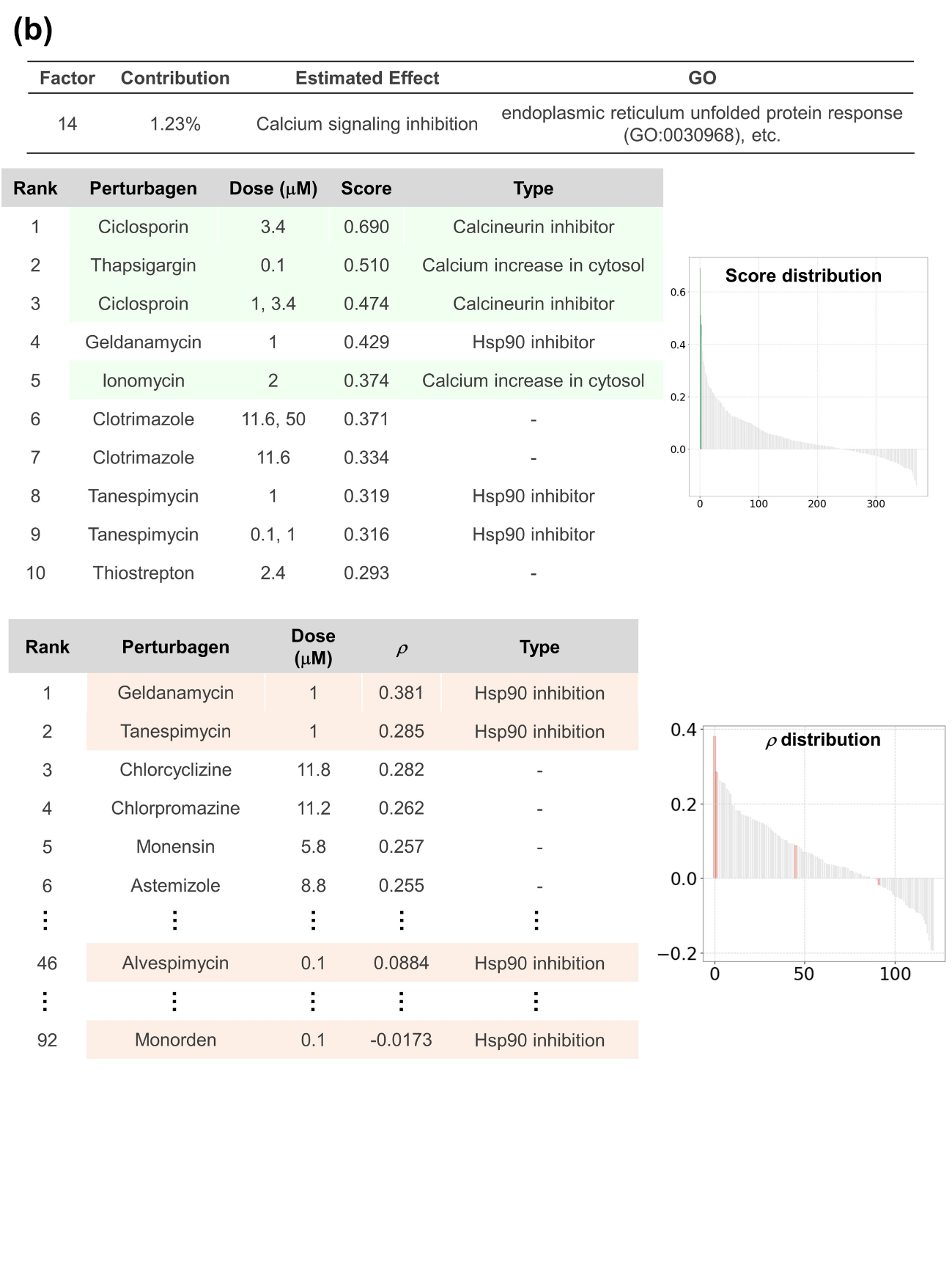


**Supplementary Fig. S5 Decomposition of Hsp90-inhibitor effect**

a. Analysis of the P22 factor using the method shown in Fig. 2c. Green or light salmon indicates Hsp90 inhibitors.

b. Analysis of P14 factor using the method shown in Fig. 2c. Green and light salmon indicate calcium signaling modulators and Hsp90 inhibitors, respectively.


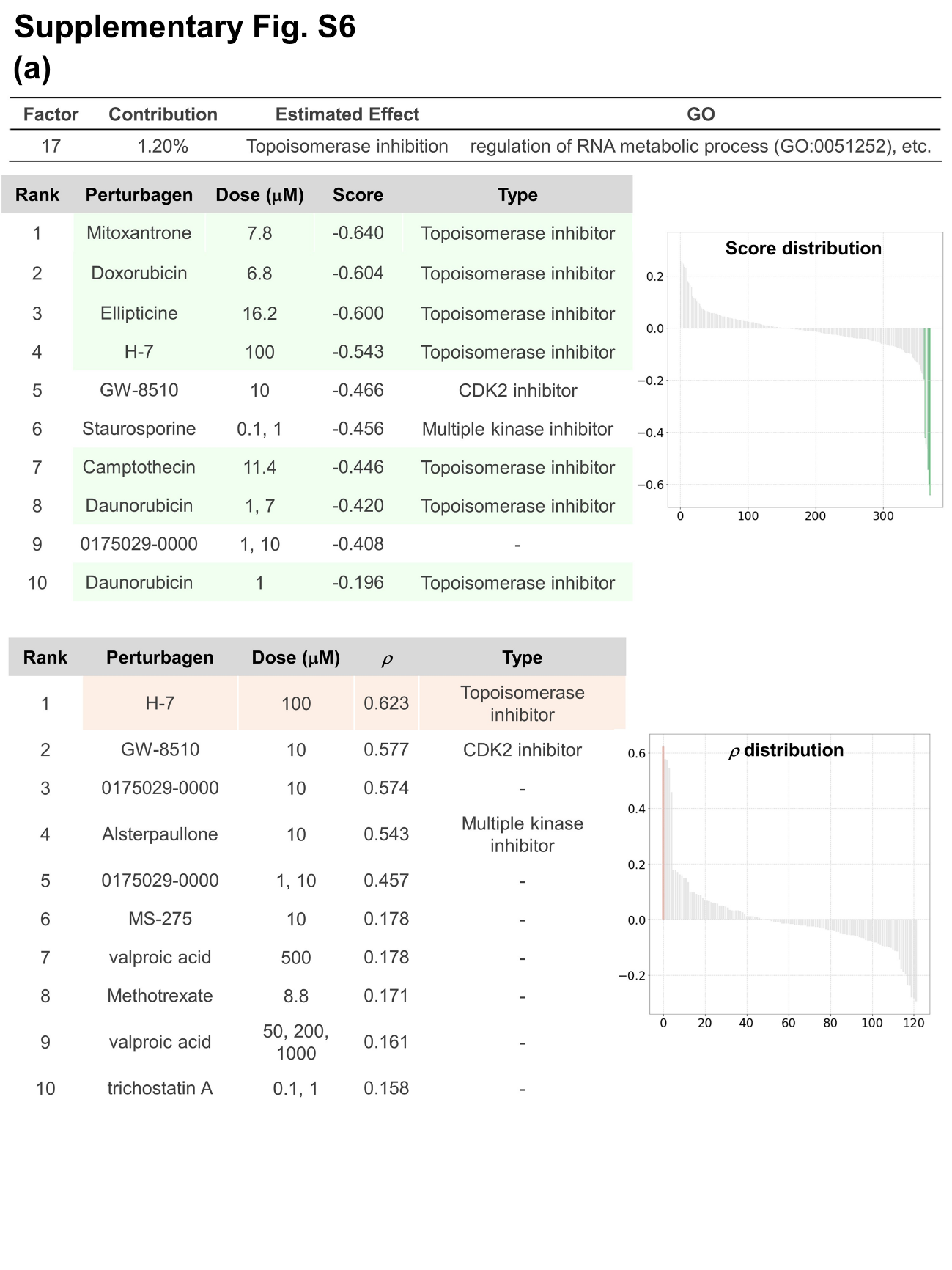


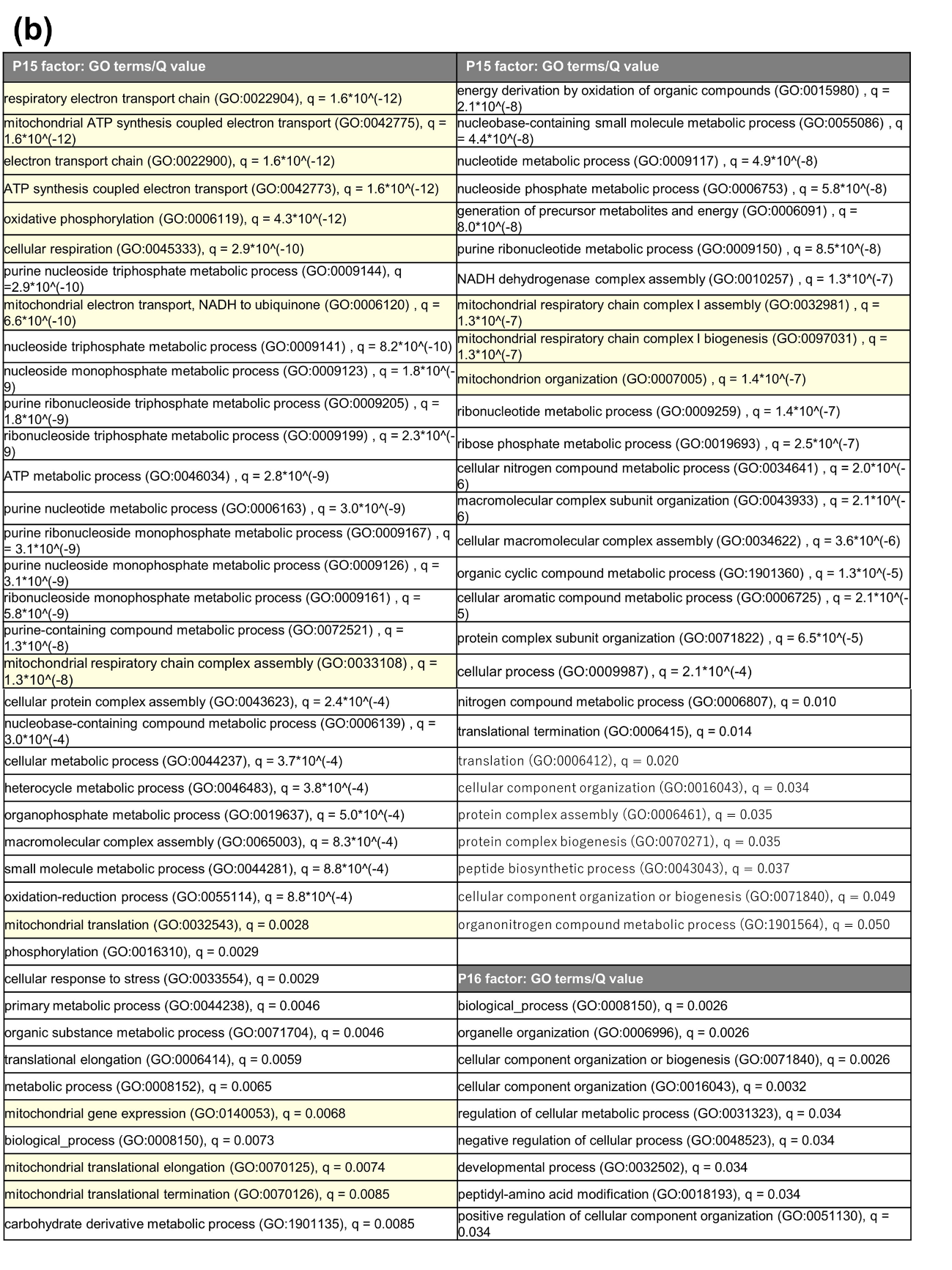


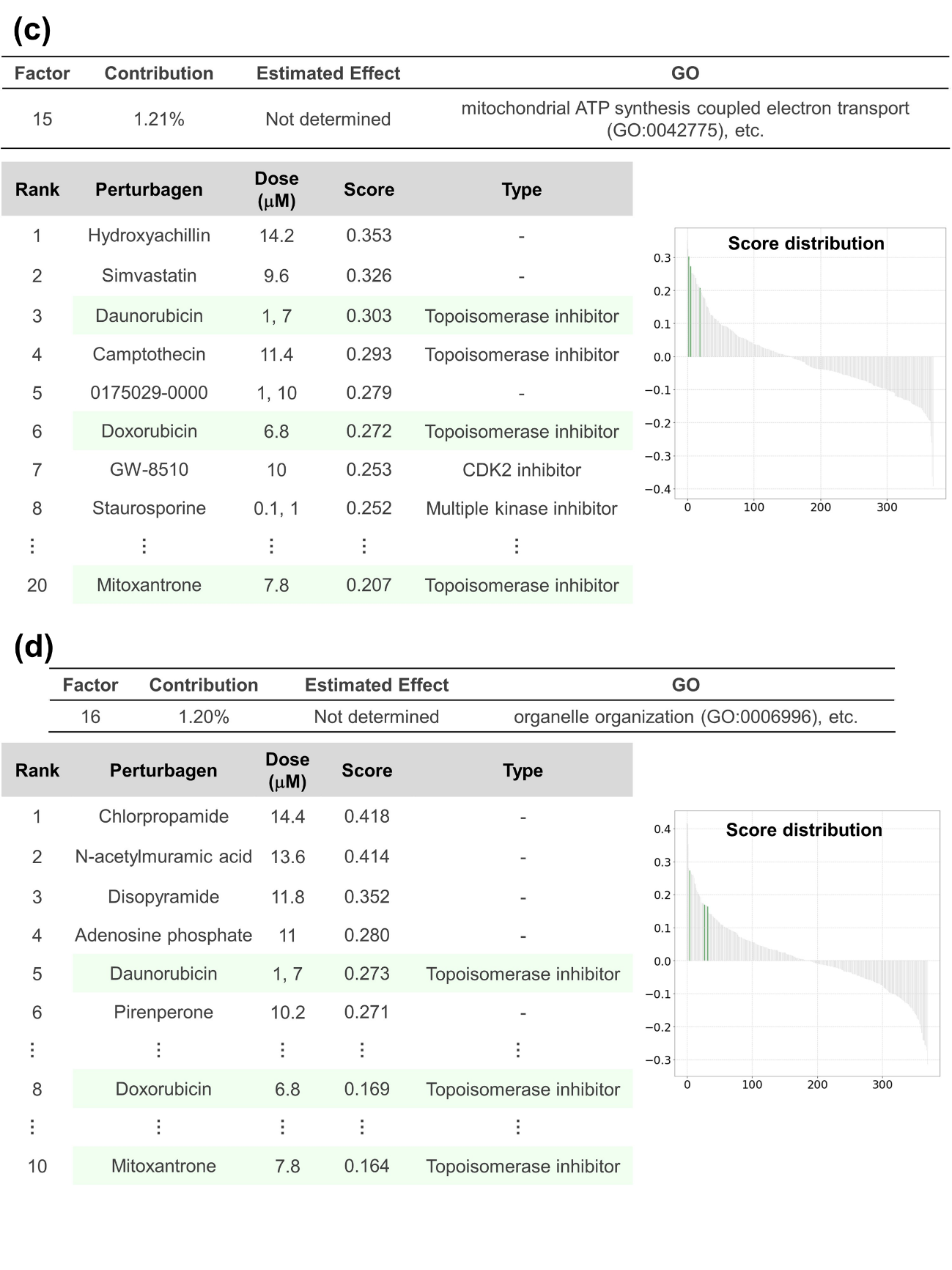


**Supplementary Fig. S6 Decomposition of topoisomerase-inhibitor effect**

a. Analysis of P17 factor using the method described in Fig 2c. Green or light salmon indicates topoisomerase inhibitors.

b. GO analysis of the P15 and P16 factors mainly constituting genes using the method described in Fig. 2b. The obtained *p*-values were processed using the Benjamini–Hochberg method for multiple-testing corrections in each factor (α < 0.05). As for the P15 factor, GO terms associated with mitochondria are filled with yellow.

c. Analysis of the P15 factor using the method described in Fig 2c. Green indicates daunorubicin, doxorubicin, and mitoxantrone.

d. Analysis of the P16 factor using the method described in Fig 2c. Green indicates daunorubicin, doxorubicin, and mitoxantrone.


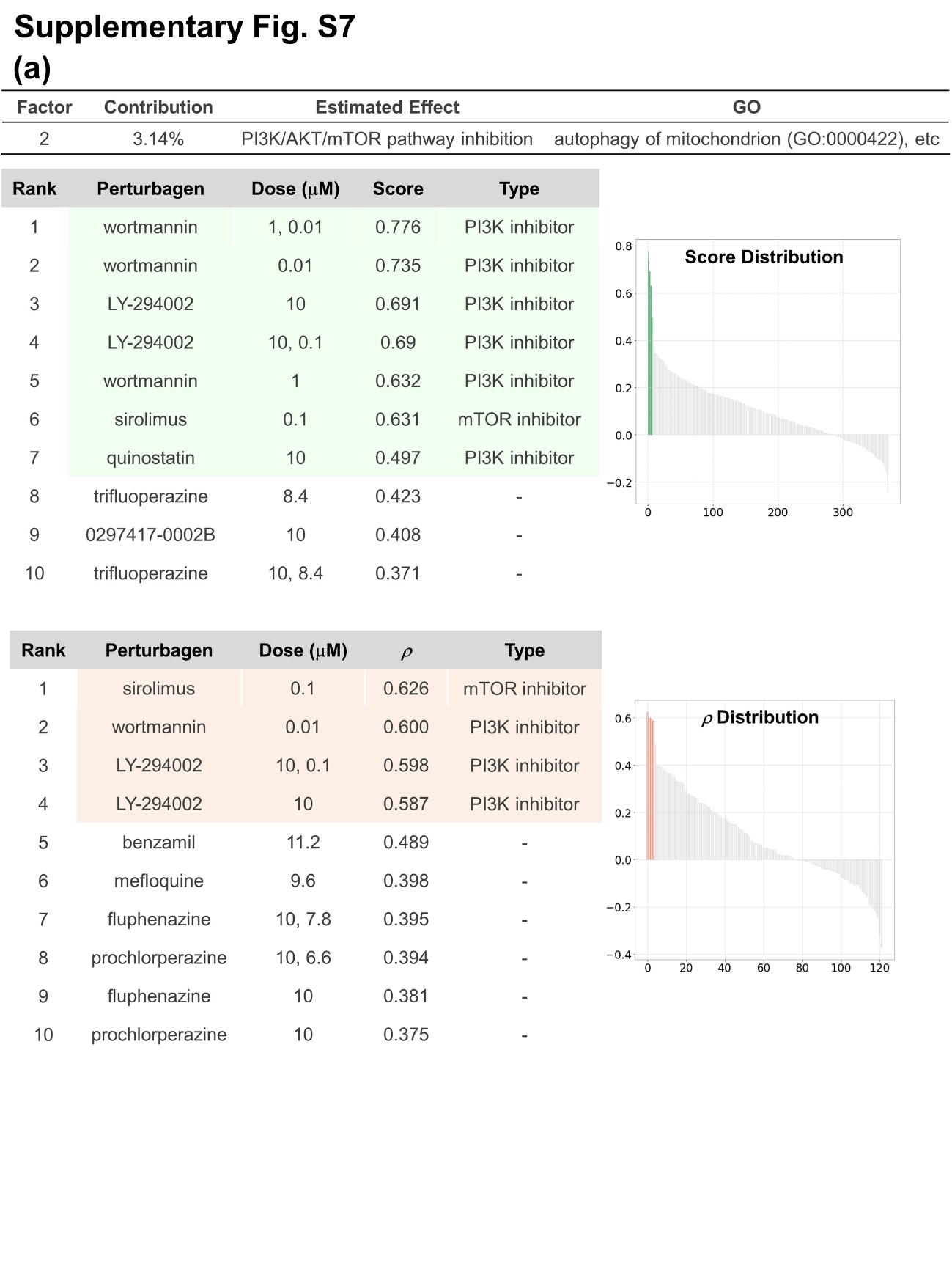


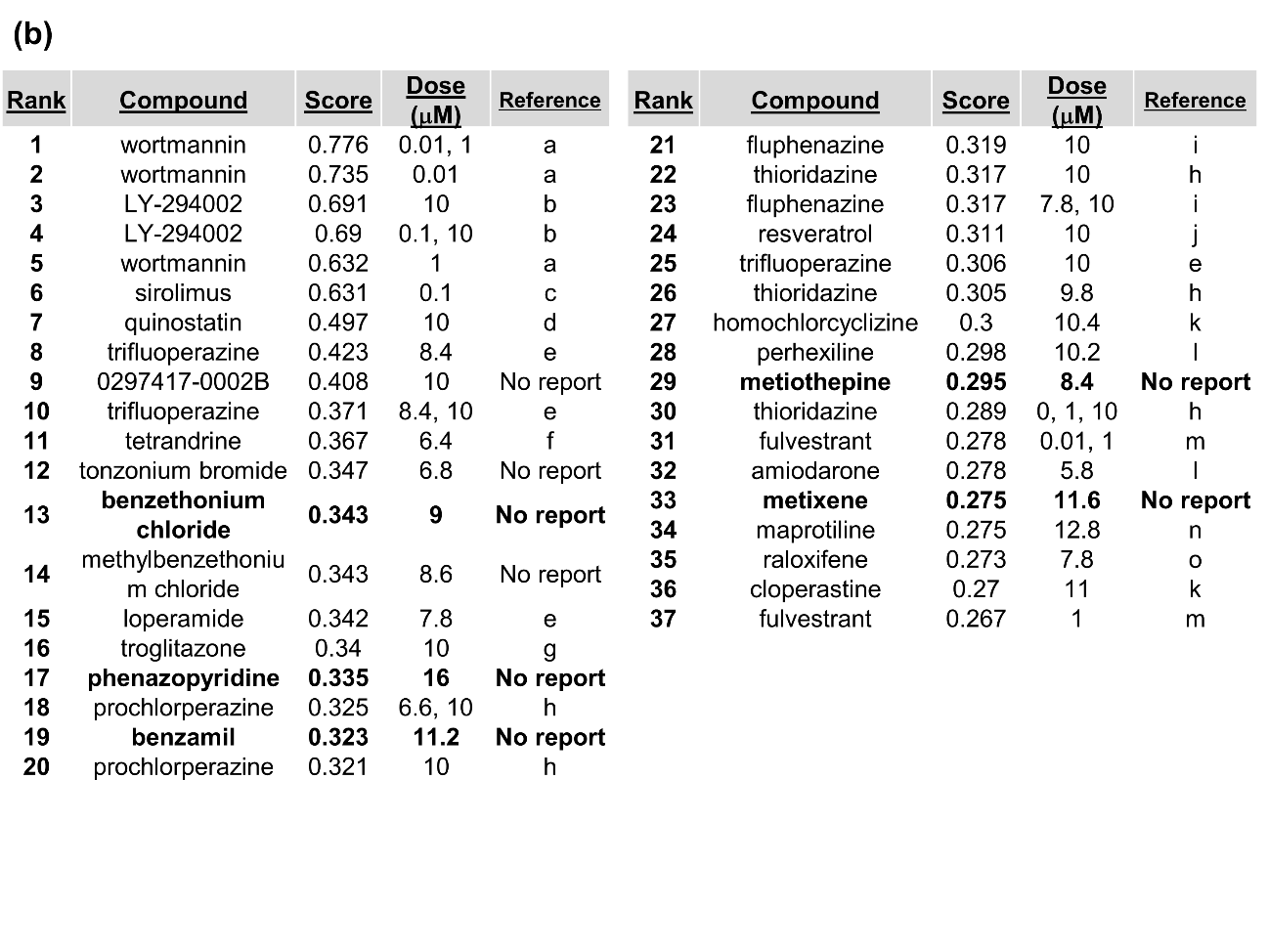


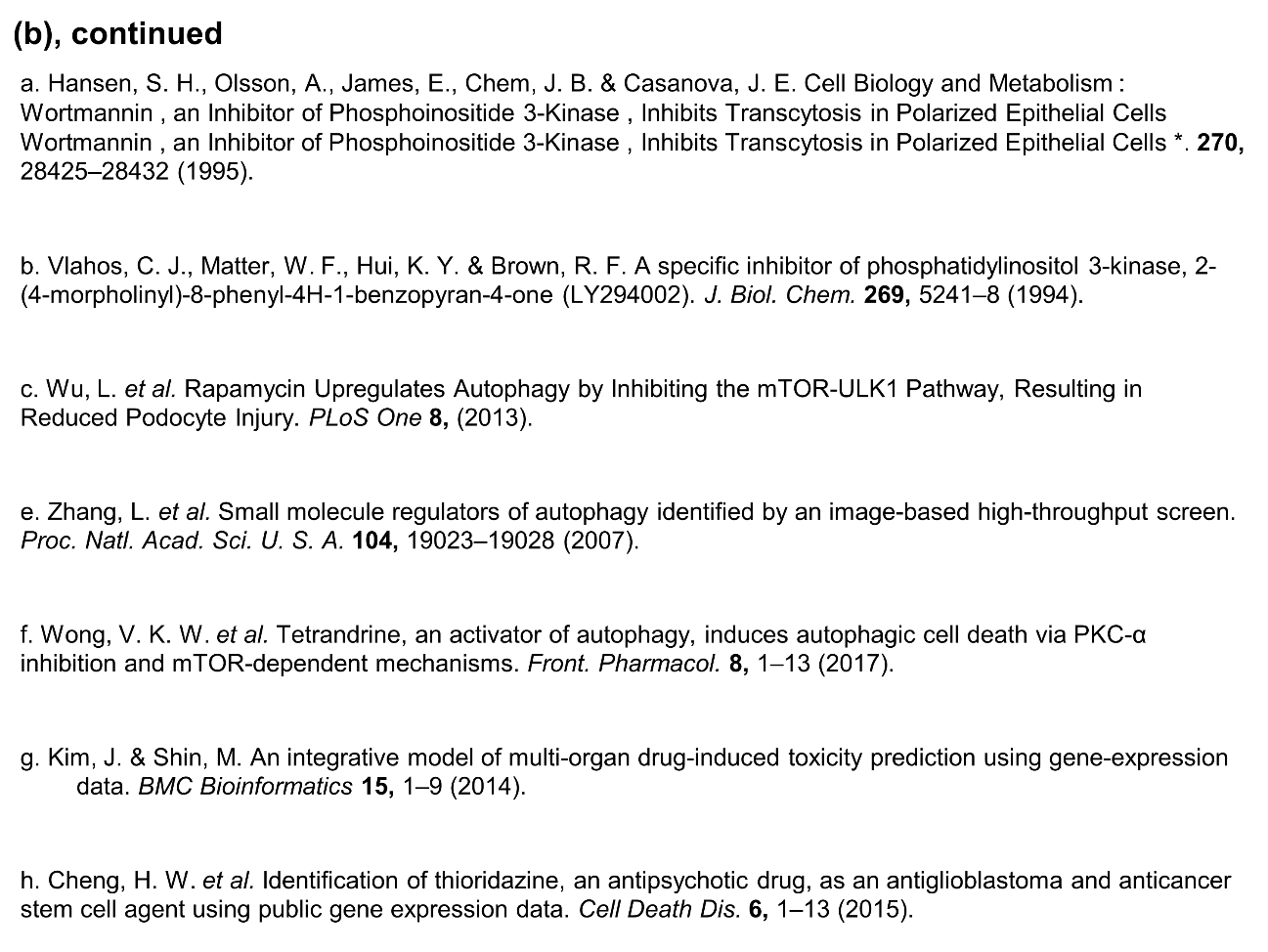


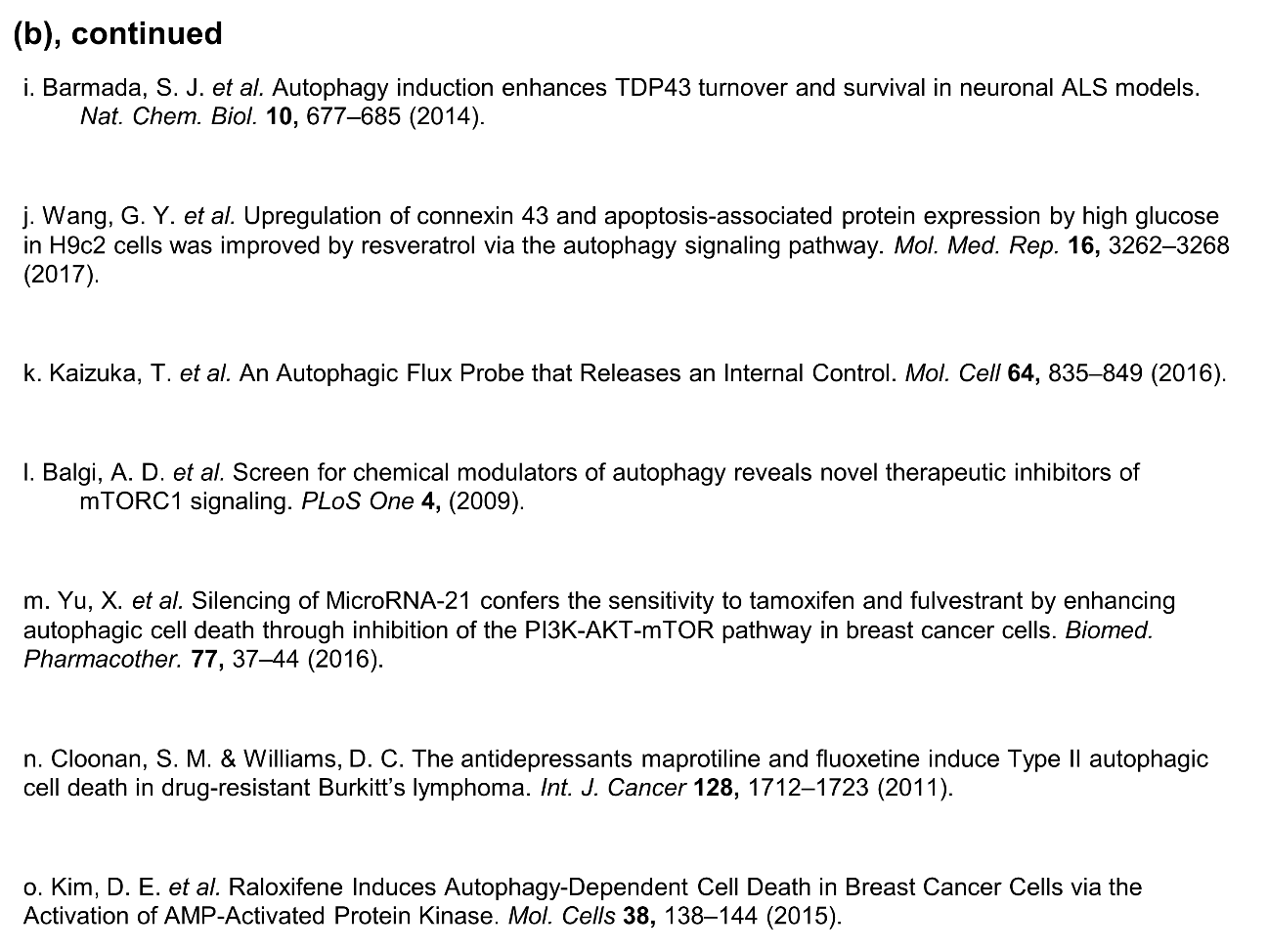


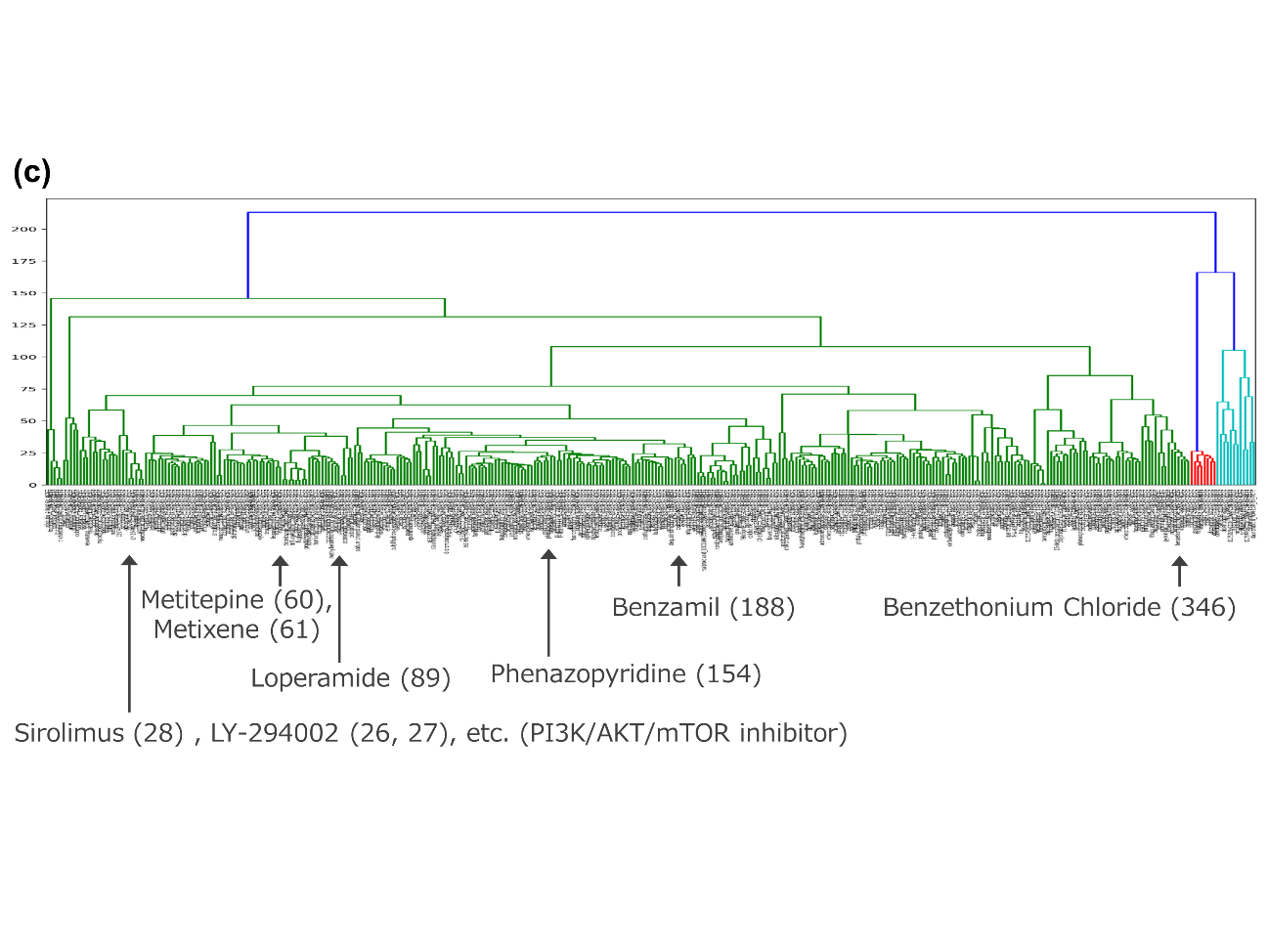


**Supplementary Fig. S7 Identification of autophagy regulator**

a. Analysis of the P2 factor using the method described in Fig. 2c. Green or light salmon indicates PI3K/Akt/mTOR pathway inhibitors.

b. List of the compounds with high scores in the P2 factor. Top 1% compounds (37) are shown.

c. Clustering analysis of MCF7 cells data set of CMap. The MCF7 cells data set of CMap was subjected to clustering analysis with the ward method. The arrow indicates the cluster where the indicated compounds belong. The numbers following the compound names indicate the ordinal numbers from the left.

d. Full-length blots of Fig. 7d. “*” indicates the bands focused on the figure.


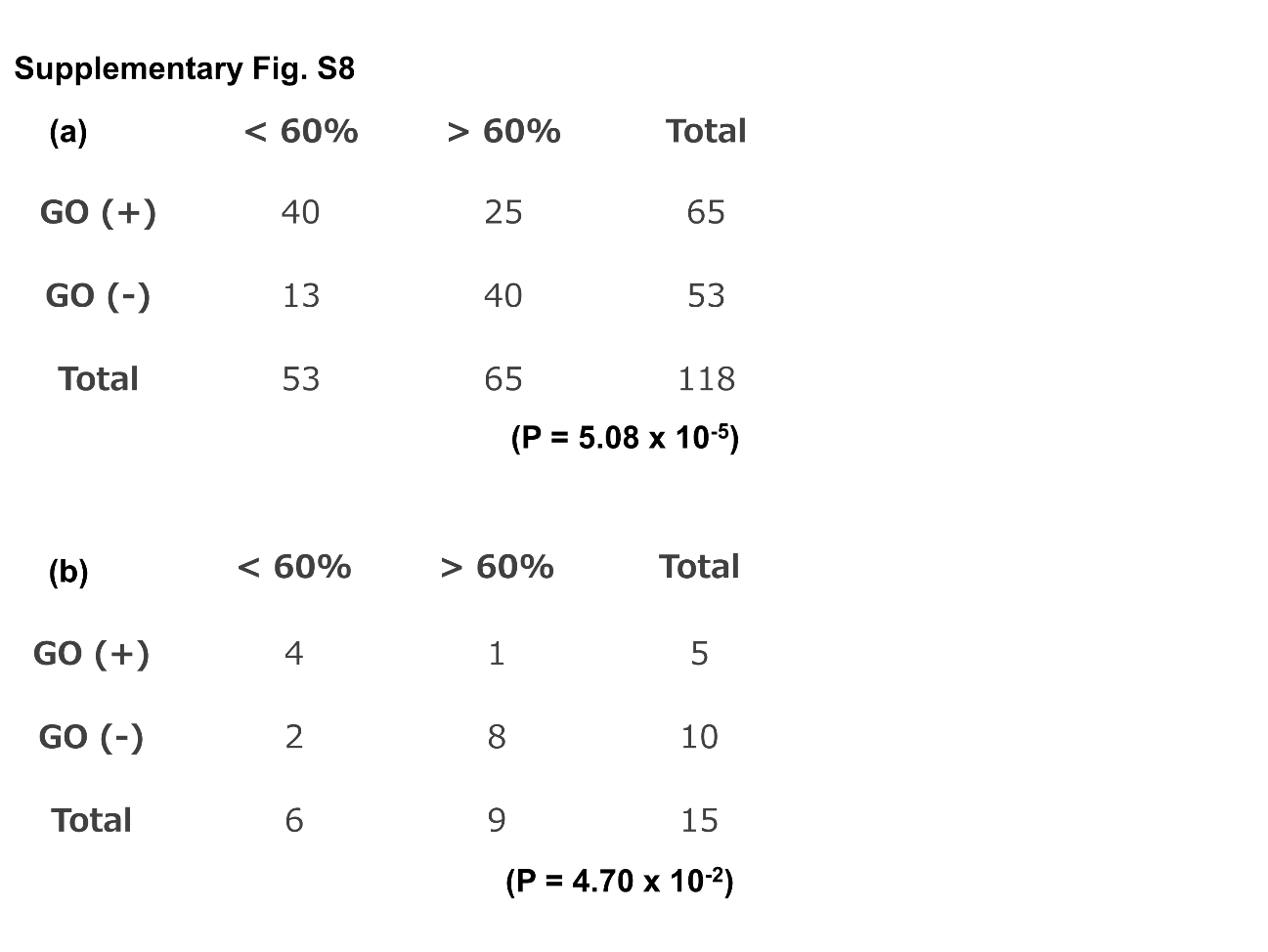


**Supplementary Fig. S8 Relationship between contribution of factors and GO**

a. A 2 × 2 contingency table for factors in CMap data set. “< 60%” and “> 60%” indicate the factors whose cumulative contribution is less and more than 60%, respectively. “GO (+)” or “GO (-)” represents the factors significantly annotated with or without GO. “P”, Fisher’s exact test probability.

b. A 2 × 2 contingency table for factors in BMDM data set. “< 60%” and “> 60%” indicate the factors whose cumulative contribution is less and more than 60%, respectively. “GO (+)” or “GO (-)” represents the factors significantly annotated with or without GO. “P”, Fisher’s exact test probability.


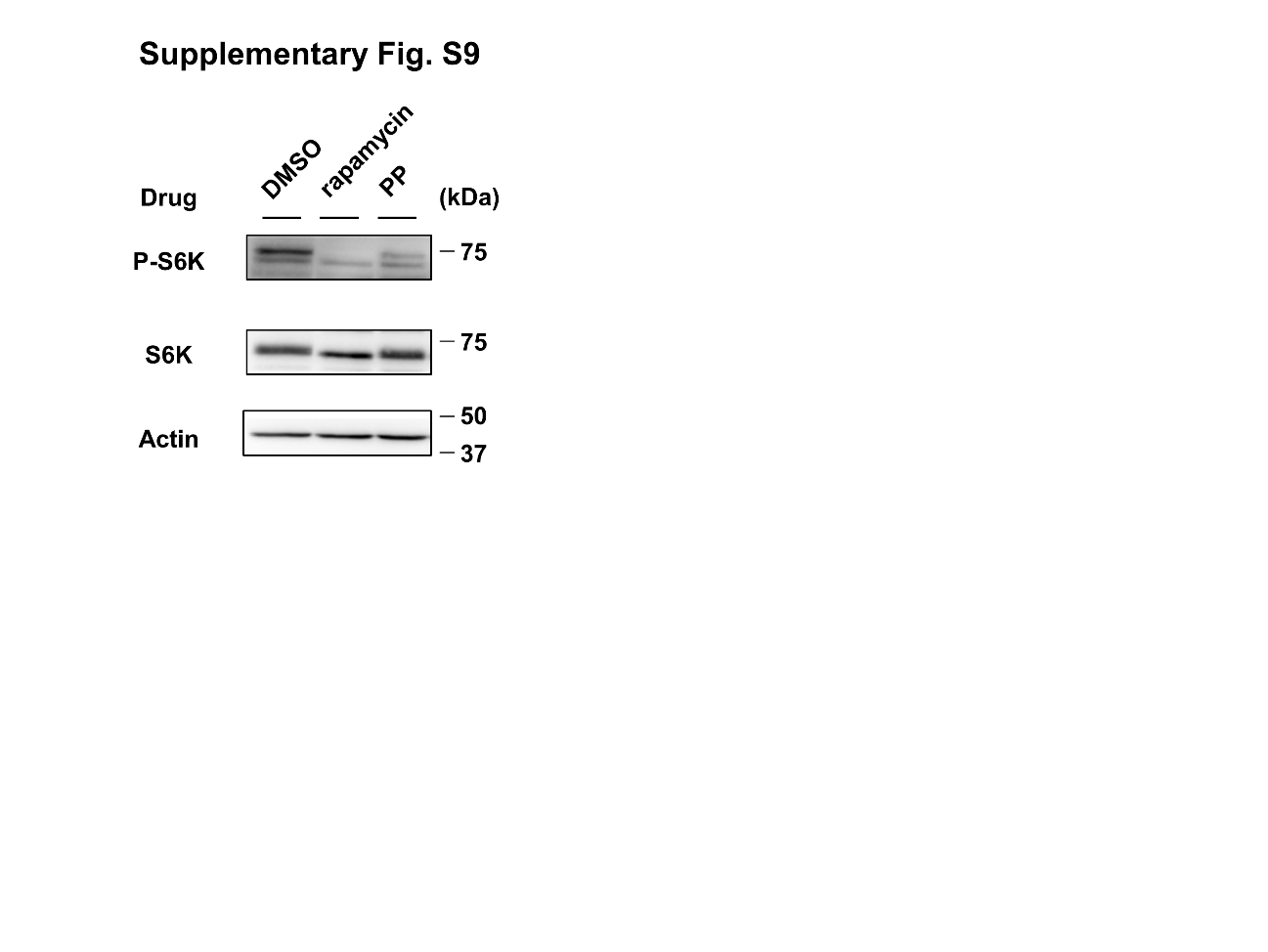


**Supplementary Fig. S9 Identification of autophagy regulator**

a. Western blotting analysis of HeLa cells treated with PP. HeLa cells were treated with PP at the indicated concentration for 6 h. Sirolimus is employed as a positive control for mTORC1 inhibition. The whole-cell lysate was analyzed by western blotting using anti-S6K and -P-S6K (Thr389) antibodies. A representative result of two independent experiments is shown. Full-length blots are presented in b.

b. Full-length blots of Fig. S9a. “*” indicates the bands focused on the figure.
