# Supplementary material for: Development of Orthogonal Linear Separation Analysis (OLSA) to Decompose Drug Effects into Basic Components
Source: Sci Rep. 2019 Feb 12;9:1824. doi: 10.1038/s41598-019-38528-4 (PMC6372619; doi:10.1038/s41598-019-38528-4)
Supplement: Supplementary file 7 — Supplementary Code [file 41598_2019_38528_MOESM7_ESM.docx]

# -*- coding: utf-8 -*-

"""

Created on Wed Jul 25 14:35:23 2018

a module for orthogonal linear separation analysis (OLSA)

@author: setsuo,shotaro, and tadahaya

"""

import sys

import csv

import math

import os

import numpy as np

import pandas as pd

np.seterr(divide='ignore', invalid='ignore')

import time

from sklearn.decomposition import PCA

from scipy import stats

from scipy.cluster.hierarchy import ward,leaves_list

class DataClass:

"""Handle data"""

def __init__(self):

self.filename=""

self.Name=list()

self.X = np.array([[],[]])

self.index = list()

def load(self,filename,read_tsv=False):

"""Load data into an object"""

self.filename=filename

print('reading file')

if read_tsv==False:

csv_obj = csv.reader(open(filename, "r"))

else:

csv_obj = csv.reader(open(filename, "r"),delimiter='\t')

data = [v for v in csv_obj]

self.Name = data[0]

#delete the 1st column

del self.Name[0]

print("sample names are")

print(self.Name)

del data[0]

data_conved = [[float(elm) for elm in v[1:]] for v in data] #Text to float

self.index = [v[0] for v in data]

self.X = np.matrix(data_conved)

print('data read OK')

def load_df(self,dataframe):

"""Load dataframe into an object"""

self.X = np.array(dataframe)

self.Name = list(dataframe.columns)

self.index = list(dataframe.index)

print('a dataframe was loaded')

def clone(self):

ret = DataClass()

ret.Name = self.Name[:] #DeepCopy

ret.X = np.array(self.X) #DeepCopy

ret.index = self.index[:] #DeepCopy

ret.filename = self.filename #DeepCopy

return ret

def deleteC(self,index):

"""delete the selected columns"""

a = range(0,len(self.Name))

b = list()

if isinstance(index, int):

b=list([index])

else:

b=index[:]

c=list(set(a)-set(b))

return self.selectC(c)

def selectC(self,index):

"""extract the selected columns"""

ret = self.clone()

ret.X = self.X[:,index]

ret.filename = self.filename

if isinstance(index, int):

ret.Name = list([self.Name[index]])

else:

ret.Name = list([self.Name[s] for s in index])

return ret

def selectR(self,index):

"""extract the selected rows"""

ret = self.clone()

ret.X = self.X[index,:]

ret.filename = self.filename

if isinstance(index, int):

ret.index = list([self.index[index]])

else:

ret.Name = list([self.Name[s] for s in index])

return ret

def bindC(X,Y):

ret = DataClass()

ret.Name = list(X.Name + Y.Name)

ret.X = np.c_[X.X,Y.X]

ret.index = list(X.index)

return ret

def wardclustersort(self,IsSpheredSort=True):

"""

sort columns according to Ward clustering

Parameters

----------

IsSpheredSort: boolean, default True

apply this function to the unit-sphereized data

"""

ret = DataClass()

ret.index = list(self.index)

ret.filename = self.filename

XS=self.X

if IsSpheredSort==True:

XS= np.array([s/np.linalg.norm(s) for s in np.array(self.X.T)]).T

sortindex = leaves_list(ward(XS.T))

ret.Name = list([self.Name[sortindex[0]]])

ret.X = self.X[:,sortindex[0]]

for i in sortindex[1:]:

ret.Name = list(ret.Name + [self.Name[i]])

ret.X = np.c_[ret.X,self.X[:,i]]

return ret

class Varimax:

"""Varimax rotation"""

def __init__(self, X):

self.n = np.shape(X)[0]

self.p = np.shape(X)[1]

self.A = np.matrix(X)

print("ok1")

if self.p > 1:

self.h = []

for i in range(0, self.n):

sum = self.A[i].dot(self.A[i].T)[0,0]

self.h.append(math.sqrt(sum))

if self.h[i]!=0:

self.A[i] /= self.h[i]

def rotateV(self,acceptable_error=1.0e-9):

"""varimax rotation"""

mm = lambda x: x*x

if (self.p < 2):

return self.A

while True:

ckA = np.matrix(self.A.A)

for i in range(0, self.p):

for j in range(i + 1, self.p):

x = np.matrix(self.A.T[i].A)

y = np.matrix(self.A.T[j].A)

u = np.matrix(x.A**2 - y.A**2)

v = np.matrix(2.0 * x.A * y.A)

cA = np.sum(u)

cB = np.sum(v)

cC = u.dot(u.T)[0,0] - v.dot(v.T)[0,0]

cD = 2.0 * u.dot(v.T)[0,0]

num = cD - 2 * cA * cB / self.n

den = cC - (mm(cA) - mm(cB)) /self.n

theta4 = math.atan(num / den)

if (num > 0.0):

if (theta4 < 0.0):

theta4 += math.pi

else:

if (theta4 > 0.0):

theta4 += math.pi

theta = theta4 / 4.0

tx = self.A.T[i] * math.cos(theta) + self.A.T[j] * math.sin(theta)

ty = -self.A.T[i] * math.sin(theta) + self.A.T[j] * math.cos(theta)

self.A.T[i] = tx

self.A.T[j] = ty

dif = np.sum((ckA.A-self.A.A)**2)

if (dif < acceptable_error):

for i in range(0, self.n):

self.A[i] *= self.h[i]

break

print("dif is %10.10f" % dif)

print(self.A[0,0])

if math.isnan(dif):

print("error")

sys.exit()

return self.A

class Result:

"""Handle OLSA results"""

def __init__(self):

self.accumulation = float()

self.sphered = bool()

self.filename=""

self.Name = list() #sample name list

self.index = list() #variable name list

self.X = np.matrix([[],[]])

self.TS = np.matrix([[],[]]) #total strength list

self.Rpca = np.matrix([[],[]]) #response vector matrix

self.Cpca=np.matrix([[],[]]) #contribution

self.AcceptNum=int()

self.NWpca = np.matrix([[],[]]) #response score matrix

self.ps =list() #vector name list

def export(self,savefilename='',TS=True,Contribution=True,RSM=True,RVM=True

,Raw=False,WxTS=False,CM=False,CMex=False,Confirmation=False):

"""

export data into a csv file

Parameters

----------

savefilename: str, default ""

a path of the output. if no description, a path is generated from the input filename

TS: boolean, default True

whether TS is exported

Contribution: boolean, default True

whether contribution is exported

RSM: boolean, default True

whether response score matrix is exported

RVM: boolean, default True

whether response vector matrix is exported

Raw: boolean, default True

whether raw is exported

WxTS: boolean, default False

whether weight x TS is exported

CM: boolean, default False

whether correlation matrix of response score is exported

CMex: boolean, default False

whether correlation matrix of response score w/o ones of

the highest and lowest vector is exported

Confirmation: boolean, default False

whether data for logical confirmation is exported

"""

def makelisttable(row,col,inputdata):

data = np.array(inputdata)

a = list(["index"] + list(col))

if len(row)>1:

rn = np.shape(data)[0]

b = [a] + list([[row[i]]+list(data[i]) for i in range(0,rn)])

else:

b = [a] + list([list(row)+list(data)])

return b

#path preparation

if len(savefilename) > 0:

basefilename = savefilename.replace('.csv','')

elif len(self.filename) > 0:

dirname = os.path.dirname(self.filename)

filename = os.path.basename(self.filename).split('.')[0]

basefilename = '{0}\\{1}'.format(dirname,filename)

else:

print('ERROR!!: No original filename or savefilename')

print('Enter savefilename in export()')

sys.exit(1)

#Saving

if TS==True:

with open('{0}_TS.csv'.format(basefilename), 'w') as f:

writer = csv.writer(f, lineterminator='\n') #indicate the newline code （\n）

writer.writerows(makelisttable([""],self.Name,self.TS))

else: pass

if Contribution==True:

with open('{0}_Cont.csv'.format(basefilename), 'w') as f:

writer = csv.writer(f, lineterminator='\n') #indicate the newline code （\n）

writer.writerows(makelisttable([""],self.ps,self.Cpca))

else: pass

if RSM==True:

with open('{0}_RSM.csv'.format(basefilename), 'w') as f:

writer = csv.writer(f, lineterminator='\n') #indicate the newline code （\n）

writer.writerows(makelisttable(self.ps,self.Name,self.NWpca))

else: pass

if WxTS==True:

with open('{0}_WxTS.csv'.format(basefilename), 'w') as f:

writer = csv.writer(f, lineterminator='\n') #indicate the newline code （\n）

wt = self.NWpca.dot(np.diag(self.TS))

writer.writerows(makelisttable(self.ps,self.Name,wt))

else: pass

if CM==True:

with open('{0}_CM.csv'.format(basefilename), 'w') as f:

writer = csv.writer(f, lineterminator='\n') #indicate the newline code （\n）

writer.writerows(makelisttable(self.Name,self.Name,np.corrcoef(self.NWpca.T)))

else: pass

if CMex==True:

with open('{0}_CMex.csv'.format(basefilename), 'w') as f:

writer = csv.writer(f, lineterminator='\n') #indicate the newline code （\n）

writer.writerows(makelisttable(self.Name,self.Name,np.corrcoef(self.NWpca.T[:,1:self.AcceptNum])))

else: pass

if RVM==True:

with open(basefilename + '_RVM.csv', 'w') as f:

writer = csv.writer(f, lineterminator='\n') #indicate the newline code （\n）

writer.writerows(makelisttable(self.index,self.ps,self.Rpca))

else: pass

if Raw==True:

with open('{0}_Raw.csv'.format(basefilename), 'w') as f:

writer = csv.writer(f, lineterminator='\n') #indicate the newline code （\n）

writer.writerows(makelisttable(self.index,self.Name,self.X))

else: pass

if Confirmation==True:

with open('{0}_Confirmation.csv'.format(basefilename), 'w') as f:

writer = csv.writer(f, lineterminator='\n') #indicate the newline code （\n）

mat = self.Rpca.dot(self.NWpca.dot(np.diag(self.TS)))

writer.writerows(makelisttable(self.index,self.Name,mat))

else: pass

print("data save finished")

def rsm(self,varimax_only=True):

"""

return response score matrix as a dataframe

"""

df = pd.DataFrame(self.NWpca)

df.index = self.ps

df.columns = self.Name

if varimax_only==True:

df2 = df.iloc[:self.AcceptNum,:]

print("return response score matrix")

else:

df2 = df

print("return response score matrix (whole vectors)")

return df2

def rvm(self,varimax_only=True):

"""

return response vector matrix as a dataframe

"""

df = pd.DataFrame(self.Rpca)

df.index = self.index

df.columns = self.ps

if varimax_only==True:

df2 = df.iloc[:,:self.AcceptNum]

print("return response vector matrix")

else:

df2 = df

print("return vector score matrix (whole vectors)")

return df2

def ts(self):

"""

return total strength as a dataframe

"""

df = pd.DataFrame(self.TS)

df.index = self.Name

df.columns = ["total strength"]

df2 = df.T

print("return total strength")

return df2

def contribution(self):

"""

return vector contribution as a dataframe

"""

df = pd.DataFrame(self.Cpca)

df.index = self.ps

df.columns = ["contribution"]

df2 = df.T

print("return contribution")

return df2

def weightedTS(self):

"""

return weighted total strength as a dataframe

"""

wt = self.NWpca.dot(np.diag(self.TS))

df = pd.DataFrame(wt)

df.index = self.ps

df.columns = self.Name

print("return weighted total strength")

return df

def cm(self):

"""

return correlation matrix as a dataframe

"""

df = pd.DataFrame(np.corrcoef(self.NWpca.T))

df.index = self.Name

df.columns = self.Name

print("return correlation matrix")

return df

def export_at_once(self,savefilename='',TS=True,Contribution=True,RSM=True,RVM=True

,Raw=True,WxTS=False,CM=False,CMex=False,Confirmation=False):

"""

export all data into a csv file

Parameters are the same with those of "export"

"""

def makelisttable(row,col,inputdata):

data = np.array(inputdata)

a = list(["index"] + list(col))

if len(row)>1:

rn = np.shape(data)[0]

b = [a] + list([[row[i]]+list(data[i]) for i in range(0,rn)])

else:

b = [a] + list([list(row)+list(data)])

return b

#path preparation

if len(savefilename) > 0: pass

else:

dirname = os.path.dirname(self.filename)

filename = os.path.basename(self.filename).split('.')[0]

savefilename = '{0}\\{1}_res.csv'.format(dirname,filename)

#Saving

with open(savefilename, 'w') as f:

writer = csv.writer(f, lineterminator='\n') #indicate the newline code （\n）

if self.sphered==False:

writer.writerow(["This data is NOT sphered."])

else:

writer.writerow(["This data is sphered."])

if TS==True:

writer.writerow("")

writer.writerow(["Total Strength"])

writer.writerows(makelisttable([""],self.Name,self.TS))

else: pass

if Contribution==True:

writer.writerow("")

writer.writerow(["Contribution of PCA"])

writer.writerow(['The number of principal components is {0}, accounting for 95% of cumulative contribution'.format(self.AcceptNum)])

writer.writerows(makelisttable([""],self.ps,self.Cpca))

else: pass

if RSM==True:

writer.writerow("")

writer.writerow(["Response Score Matrix"])

writer.writerows(makelisttable(self.ps,self.Name,self.NWpca))

else: pass

if WxTS==True:

writer.writerow("")

writer.writerow(["Weight * diag(TS) for graphical modeling"])

wt = self.NWpca.dot(np.diag(self.TS))

writer.writerows(makelisttable(self.ps,self.Name,wt))

else: pass

if CM==True:

writer.writerow("")

writer.writerow(["Correlation of Response Score Matrix"])

writer.writerows(makelisttable(self.Name,self.Name,np.corrcoef(self.NWpca.T)))

else: pass

if CMex==True:

writer.writerow("")

writer.writerow(["Correlation of Response Score Matrix Excluding First and Minor Components"])

writer.writerow(self.Name)

writer.writerows(makelisttable(self.Name,self.Name,np.corrcoef(self.NWpca.T[:,1:self.AcceptNum])))

else: pass

if RVM==True:

writer.writerow("")

writer.writerow(["Response Vector Matrix"])

writer.writerows(makelisttable(self.index,self.ps,self.Rpca))

else: pass

if Raw==True:

writer.writerow("")

writer.writerow(["Raw Data"])

writer.writerows(makelisttable(self.index,self.Name,self.X))

else: pass

if Confirmation==True:

writer.writerow("")

writer.writerow(["Reponse * Weight * diag(TS) for logical confirmation"])

mat = self.Rpca.dot(self.NWpca.dot(np.diag(self.TS)))

writer.writerows(makelisttable(self.index,self.Name,mat))

else: pass

print("data save finished")

class SmirnovGrubbs:

def __init__(self):

self.RemainedIndexes = list()

self.RemovedIndexesHigher = list()

self.RemovedIndexesLower = list()

self.NonbiasedVar = float()

self.alpha = float()

def calcSG(TS,alpha):

"""conduct SG test to exclude the data with unusual TS"""

res = SmirnovGrubbs()

res.alpha = alpha

Data = list()

RemovedDataHigher = list()

RemovedDataLower = list()

for i in range(0,TS.shape[0]):

Data.append([i,TS[i]])

while True:

n=len(Data)

if n<3:

break

t = stats.t.isf((alpha/n) / 2, n-2)

Gtest = (n-1)/math.sqrt(n) * math.sqrt(t**2 / (n-2 + t**2))

mean=0.0

for d in Data:

mean = mean + d[1]

mean = mean / n

var = 0.0

for d in Data:

var = var + (d[1]-mean)*(d[1]-mean)

var = var / n

sd = math.sqrt(var)

maxindex = Data[0][0]

maxvalue = math.fabs(Data[0][1] - mean)

for i in range(0,len(Data)):

if maxvalue < math.fabs(Data[i][1] - mean):

maxindex = i

maxvalue = math.fabs(Data[i][1] - mean)

#SmirnovGrubbs

if maxvalue / sd > Gtest:

if (Data[maxindex][1]-mean)>0:

RemovedDataHigher.append([Data[maxindex][0],Data[maxindex][1]])

else:

RemovedDataLower.append([Data[maxindex][0],Data[maxindex][1]])

del Data[maxindex]

else:

break

mean=0.0

for d in Data:

mean = mean + d[1]

mean = mean / n

ubvar = 0.0

for d in Data:

ubvar = ubvar + (d[1]-mean)*(d[1]-mean)

ubvar = ubvar / (n-1.0)

res.NonbiasedVar = ubvar

for d in Data:

res.RemainedIndexes.append(int(d[0]))

for d in RemovedDataHigher:

res.RemovedIndexesHigher.append(int(d[0]))

for d in RemovedDataLower:

res.RemovedIndexesLower.append(int(d[0]))

return res

def usspca(Data,accumulation=0.95,IsSphered=True,

UseMirror=True,UseSmirnovGrubbs=True,WardClusterSort=True):

"""

conduct Unit Spherized Symmetric PCA

Parameters

----------

Data: DataClass object

subjected to analysis

accumulation: float, default 0.95

% of cumulative contribution of the calculated vectors

IsSphered: boolean, default True

whether data is unit-sphereized before calculation

UseMirror: boolean, default True

whether the input data set is combined with the origin-symmetric set before calculation

UseSmirnovGrubbs: boolean, default True

whether outliers are excluded according to SG test

WardClusterSort: boolean, default True

whether response score matrix is sorted according to clustering with ward method

"""

if WardClusterSort==True: Data2 = Data.wardclustersort()

else: Data2 = Data

res = Result()

res.accumulation = accumulation

res.sphered = IsSphered

res.Name = Data2.Name[:]

res.index = Data2.index[:]

res.X = np.array(Data2.X)

res.filename = Data2.filename

print ('********************************')

print ('The conditions of this proccess')

print(('Contribution Accumulation Max is ' + str(accumulation)))

print(('IsSphered Flag is ' + str(IsSphered)))

print(('UseMirror Flag is ' + str(UseMirror)))

print(('UseSmirnovGrubbs Flag is ' + str(UseSmirnovGrubbs)))

print ('********************************')

#store Total Strength

res.TS = np.array([np.linalg.norm(s) for s in res.X.T])

if UseSmirnovGrubbs==True:

SGres = calcSG(res.TS,0.05)

print("excluded samples by SG test")

print("too large norm：")

print(list([res.Name[s] for s in SGres.RemovedIndexesHigher]))

print("too small norm：")

print(list([res.Name[s] for s in SGres.RemovedIndexesLower]))

#data reconstruction

remainedX = np.array([res.X.T[s] for s in SGres.RemainedIndexes]).T

else:

#data reconstruction

remainedX = res.X

#map the data on a unit sphere

XS = np.array(res.X)

remainedXS = np.array(remainedX)

if res.sphered==True:

XS = np.array([s/np.linalg.norm(s) for s in res.X.T]).T

remainedXS = np.array([s/np.linalg.norm(s) for s in remainedX.T]).T

#add a mirror data set origin-symmetric to the analyzed set

#in order to approximate the centroid to the origin

if UseMirror==True:

Xpca =np.c_[remainedXS, remainedXS*-1]

else:

Xpca =np.c_[remainedXS]

#calculate the centroid of the data set

#np.c_[] for conversion into nx1 vector

Centroid = np.c_[np.array([sum(s)/len(s) for s in Xpca])]

print('data preparation OK')

##PCA

decomposer = PCA()

decomposer.fit(Xpca.T)

print('PCA calculation OK')

#Response Profile Matrix

SampleNum = np.shape(remainedX)[1]

res.Rpca = decomposer.components_.T [:,0:SampleNum]

#Contributaion Vector

res.Cpca=decomposer.explained_variance_ratio_[0:SampleNum]

res.ps =['%s%d' % ('P',s) for s in range(1,len(res.Cpca)+1)] #label like p1~

AccumuC = 0.0

res.AcceptNum=0

for i in range(0,len(res.Cpca)):

res.AcceptNum = res.AcceptNum+1

AccumuC = AccumuC + res.Cpca[i]

if AccumuC > res.accumulation:

break

print("The selected components number is %d" % res.AcceptNum)

print("The accumulation value is %f" % AccumuC)

#Normalized Weight Matrix

#subtract the centroid from the input data in PCA

#as for OLSA, the centroid matches the origin

res.NWpca = res.Rpca.T.dot(XS-Centroid)

return res

def olsa(Data,accumulation=0.80,FixSet=set(),IsSphered=True,

UseMirror=True,UseSmirnovGrubbs=True,acceptable_error=1.0e-9,WardClusterSort=True):

"""

conduct orthogonal linear separation analysis (OLSA) and return a Result object

Parameters

----------

Data: DataClass object

subjected to analysis

accumulation: float, default 0.80

% of cumulative contribution of the vectors subjected to varimax rotation

FixSet: int

indicates the column number not subjected to varimax rotation

IsSphered: boolean, default True

whether data is unit-sphereized before calculation

UseMirror: boolean, default True

whether the input data set is combined with the origin-symmetric set before calculation

UseSmirnovGrubbs: boolean, default True

whether outliers are excluded according to SG test

acceptable_error: float, default 1.0e-9

determines acceptable error in varimax rotation

WardClusterSort: boolean, default True

whether response score matrix is sorted according to clustering with ward method

"""

start = time.time()

res = usspca(Data,accumulation,IsSphered,UseMirror

,UseSmirnovGrubbs,WardClusterSort)

#store filename

res.filename = Data.filename

XS = np.array(res.X)

if IsSphered==True:

XS = np.array([s/np.linalg.norm(s) for s in res.X.T]).T

#subject the vectors accounting for *% of cumulative contribution to varimax rotation

#some vectors can be fixed if indicated

Vselect = set(range(0,res.AcceptNum))

if isinstance(FixSet, int):

Vselect = set(Vselect)-set([FixSet])

else:

Vselect = set(Vselect)-set(FixSet)

Vtotal = set(range(0,np.shape(res.Rpca)[1]))

VResidue = Vtotal - Vselect

print("Vselect = " , Vselect)

print("VResidue = " , VResidue)

ForVarimaxData = res.Rpca[:,list(Vselect)]

for i in list(Vselect):

res.ps[i] = "%sV" % res.ps[i]

res.ps = [res.ps[i] for i in list(Vselect)+list(VResidue)]

print("Call Varimax class ")

rot = Varimax(ForVarimaxData)

rotated = np.array(rot.rotateV(acceptable_error))

#Sort of rotated

SeudoNW=rotated.T.dot(XS)

SeudoVars = np.array([s.var() for s in np.c_[SeudoNW,SeudoNW* -1]])

Slist=list()

for i in range(0,len(SeudoVars)):

Slist.append([SeudoVars[i],i])

#ascending

Slist.sort()

#reverse the ranking

Slist.reverse()

Srotated=np.array(rotated)

for i in range(0,len(Slist)):

Srotated[:,i]=rotated[:,Slist[i][1]]

###loading matrix

res.Rpca = np.c_[Srotated,res.Rpca[:,list(VResidue)]]

print("Calc New NWpca")

###loading matrix

res.NWpca = res.Rpca.T.dot(XS)

print("Varimax Finished")

#calculate the contribution ratio

Vars = np.array([s.var() for s in np.c_[res.NWpca,res.NWpca * -1]])

res.Cpca = Vars/Vars.sum()

#display elapsed time

passed = time.time() - start

h,m = divmod(passed,3600)

m,s = divmod(m,60)

print('elapsed time: {0} hr {1} min {2} sec'.format(int(h),int(m),round(s,-2)))

print("return a Result object")

return res
